# Discovery of Fragment-Based Inhibitors of SARS-CoV‑2 PL^Pro^

**DOI:** 10.1021/acs.jmedchem.5c02832

**Published:** 2026-01-12

**Authors:** Qiangqiang Wei, Ashley J. Taylor, Mahesh Angadrao Barmade, Kevin B. Teuscher, Somanath Chowdhury, Chideraa Apakama, Jordan Anderson-Daniels, Zhu Yongqing, David C. Schultz, Tyson A. Rietz, Taylor M. South, Mackenzie M. Crow, Bin Zhao, Kangsa Amporndanai, John L. Sensintaffar, Jason Phan, Sara Cherry, Mark Denison, Taekyu Lee, Stephen W. Fesik

**Affiliations:** † Department of Biochemistry, 12327 Vanderbilt University School of Medicine, Nashville, Tennessee 37232-0146, United States; ‡ Department of Pathology, Microbiology, and Immunology, 5718 Vanderbilt University Medical Center, Nashville, Tennessee 37232, United States; § Department of Pathology and Laboratory Medicine, 6572 University of Pennsylvania, Philadelphia, Pennsylvania 19104, United States; ∥ Department of Pharmacology, 12327 Vanderbilt University School of Medicine, Nashville, Tennessee 37232-6600, United States; ⊥ Department of Chemistry, Vanderbilt University, Nashville, Tennessee 37235, United States

## Abstract

SARS-CoV-2 papain-like protease (PL^Pro^) plays a key role in viral replication and the host immune response and is a promising target for developing new antiviral treatments. We previously reported a fragment-based screen to identify hits that bind to SARS-CoV-2 PL^Pro^. Here, we describe the discovery of potent PL^Pro^ inhibitors by optimizing one of these hits via extensive medicinal chemistry guided by multiple X-ray structures of cocomplexes. Lead compound **46** is shown to bind to the S3 and S4 pockets with nanomolar affinity (0.4 μM) and exhibits robust cellular activity and resistance to mutation. This novel class of PL^Pro^ inhibitors can potentially be used as a starting point for the development of inhibitors to combat the emergence of drug-resistant viral strains and future coronavirus outbreaks.

## Graphical abstract

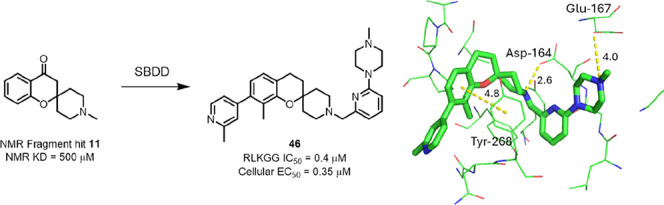

## Introduction

Over the past two decades there have been several viral outbreaks caused by members of the coronavirus family^1–3^ the largest being the COVID-19 pandemic of 2019–2022 which claimed over 7 million lives worldwide.^4,5^ Given the high prevalence and pathogenicity of this viral family it is critical to develop antiviral drugs that target key proteins in the coronavirus life cycle. Currently only 3 drugs have been approved by the FDA for the treatment of Covid-19: two RNA dependent RNA polymerase inhibitors (Remdesivir and Molnupiravir)^6,7^ and the main protease inhibitor Nirmatrelvir,^8^ all of which have significant drawbacks and limitations to their application. Most concerning is the rise of drug resistant mutants which have been observed in both the laboratory and clinical settings,^9,10^ highlighting the need to develop drugs with novel mechanisms of action.

Following replication, the SARS-CoV-2 viral polyprotein is cleaved by two cysteine proteases, the papain-like protease (PL^Pro^) and 3CL main protease (M^Pro^), which cleave nonstructural proteins 1–3 and 4–16, respectively.^11–13^ Even though both proteases are essential for viral replication, only the M^Pro^ inhibitor Nirmatrelvir has been approved against SARS-CoV-2. As of yet, no PL^Pro^ inhibitors have progressed to clinical trials. In addition to its role in the viral replication cycle, PL^Pro^ also aids in the evasion of the host immune response through the cleavage of the ubiquitin and interferon-stimulated gene 15.^14–17^ These two factors paired with the high homology of PL^Pro^ across members of the coronavirus family suggests that PL^Pro^ is a promising target for the development of novel drugs against coronavirus infection.

One reason for the lack of PL^Pro^ inhibitors is the nature of the PL^Pro^ active site, which makes it difficult to identify potent inhibitors of this enzyme.^18^ The substrate specificity of PL^Pro^ (RLXGG)^19,20^ means the S1 and S2 subsites form a narrow tunnel blocking access to the catalytic triad, requiring ligands to bind in the largely solvent exposed S3 and S4 subsites (Figure 2). In addition, one side of the active site binding pocket is formed by the highly flexible loop (BL2),^21,22^ which adds to the difficulty in identifying suitable chemical scaffolds to begin a drug discovery effort. Despite multiple drug discovery campaigns against PL^Pro^ all reported inhibitors–both irreversible and reversible–have been derived from a single chemical scaffold (Figure 1).^23–26^ GRL-0617 was first reported as an inhibitor of SARS-CoV-1 PL^Pro^ in 2008.^27^ Due to the high sequence homology of the coronavirus family, GRL-0617 was also found to inhibit SARS-CoV-2 PL^Pro^. Multiple groups have attempted to develop GRL-0617 derived inhibitors of PL^Pro^; however, none have progressed to clinical trials. Irreversible analogues of GRL-0617 with the inclusion of a hydrazine diamide linker to protrude through the glycine channel resulted in poor pharmacokinetic profiles and limited drug permeability.^26^ Reversible inhibitors developed by Rutgers University and the University of Illinois showed that expansion into the BL2 groove and S3 subsite could further increase the potency of GRL-0617, but analogues still had relatively low cellular activity requiring high doses (200–500 mpk BID) to see a pharmacological effect.^23,24^ Pfizer has also developed GRL-0617 analogues with higher potency that exhibited in vivo efficacy validating PL^Pro^ as anticoronaviral target.^25^ Multiple GRL-0617 derived inhibitors have proven the therapeutic viability of PL^Pro^; however, their failure to reach the clinic highlights the need to develop a novel class of inhibitors for this enzyme.

**Figure 1:**
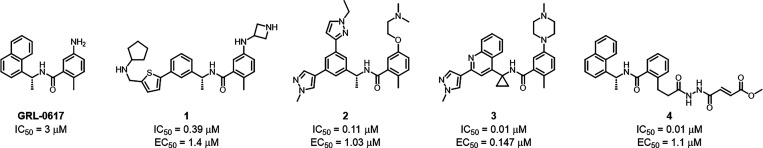
Structure of the first reported PL^Pro^ inhibitor GRL-0617 ^27^ and its analogues developed by University of Illinois **1**,^24^ Rutgers University **2**,^23^ Pfizer **3**,^25^ and Oak Ridge National Laboratory **4** ^26^ with their inhibitory and cellular activity reported.

**Figure 2:**
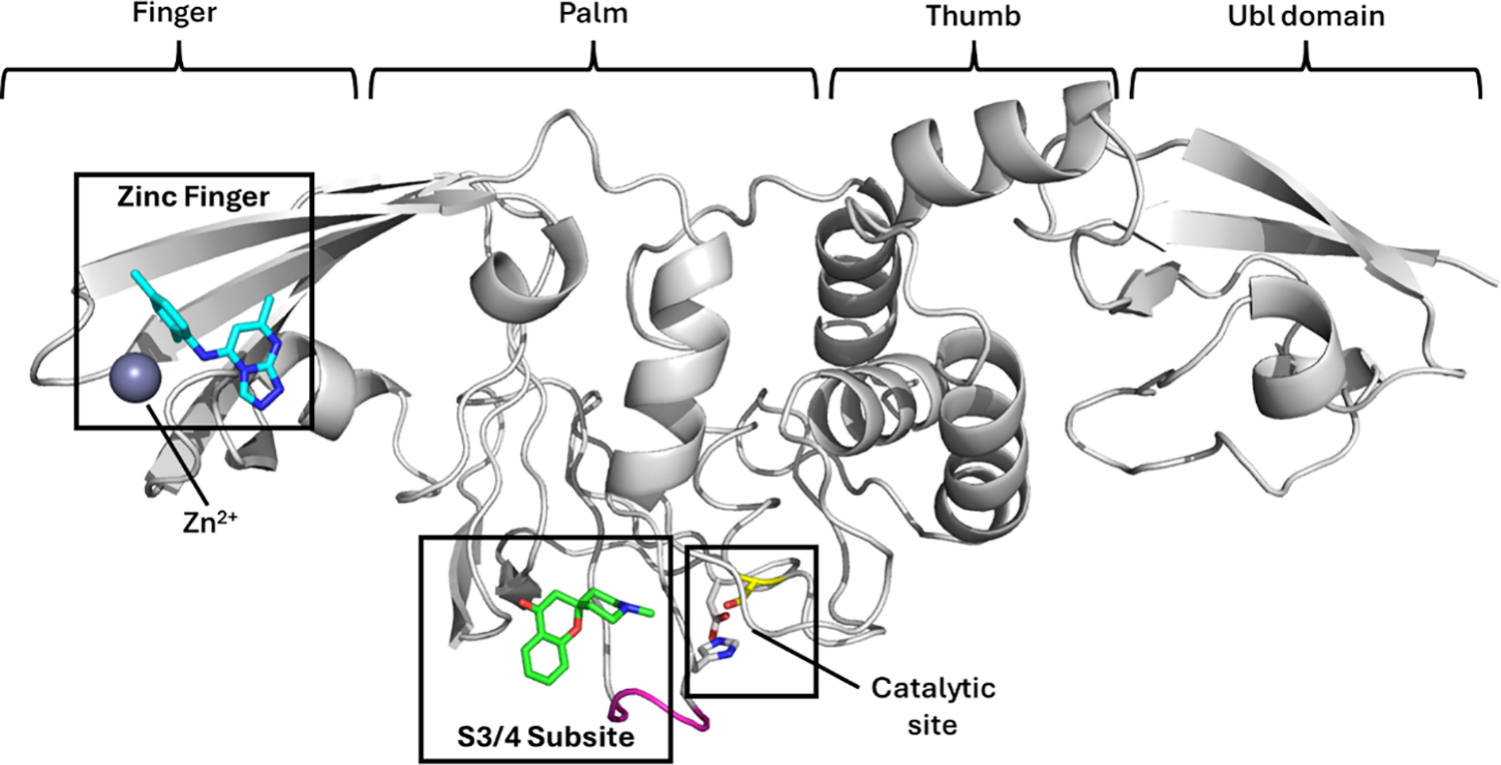
X-ray crystal structure of PL^Pro^ with previously reported zinc finger binder^28^ (blue, PDB 9BRX) and lead fragment hit **11** (green, PDB 9PUH) shown as sticks. Catalytic triad shown as sticks with active Cys-111 colored yellow and flexible BL2 loop of the S3/4 subsites colored in purple.

We have previously reported on a fragment-based screen of PL^Pro^.^28^ Here we utilize structure-based design to optimize a key fragment obtained in the screen to achieve submicromolar inhibitors of SARS-CoV-2 PL^Pro^. These inhibitors demonstrate robust antiviral cellular activity and represent a good starting point for the development of clinically useful SARS-CoV-2 inhibitors with a different scaffold than that of GRL-0617.

## Results and Discussion

### Initial Fragment Selection and Optimization

In our previously reported fragment screen against SARS-CoV-2 PL^Pro^ using a fragment library containing 13,824 compounds, 77 fragments were identified that bound to two distinct binding regions located at the S3 and S4 pockets adjacent to the active site and at the zinc finger domian.^28^ While fragments bound at the zinc finger domain showed clear SAR trends and avenues for expansion, they failed to show activity in an enzymatic inhibition assay. Several classes of fragments were found to bind at the S3 and S4 pockets with *K*_D_’s ranging from 2 mM to 380 μM (Figure 3). All fragments that bound to the S3 and S4 pockets showed activity in the enzymatic inhibition assay. Due to the clear SAR trends and avenues for expansion the spiro chromanone fragment series exemplified by **10** and **11** was selected for further elaboration.

**Figure 3:**
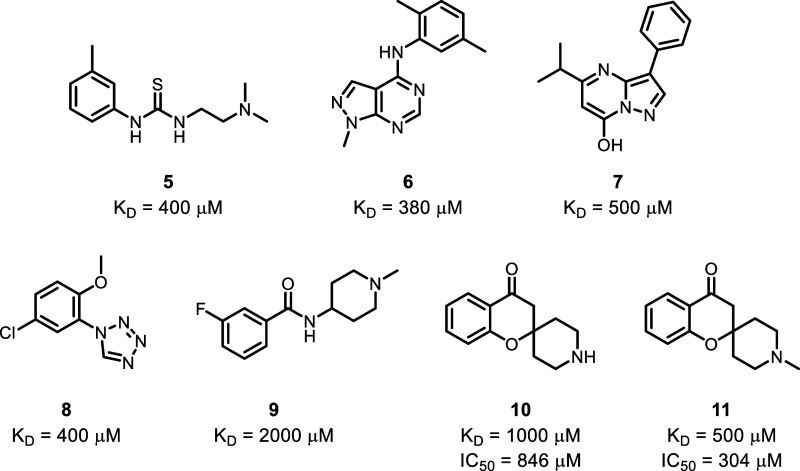
Structure of fragments from various chemical classes were found to bind at the S3 and S4 pockets of SARS-CoV-2 PL^Pro^ with their K_D_’s (determined by NMR HMQC titrations) and IC_50_’s (determined by biochemical inhibition assay) reported.

Both the initial fragment hits **10** and **11** showed submillimolar binding by NMR and enzymatic inhibition assays with the *N*-methyl being ∼3-fold more active than the free amine. Initial efforts were focused on the optimization of the fragment core. Screening of commercially available and in house analogues suggested that the 7 and 8-positions of the phenyl ring were most tolerant of substitution (Table 1). Introduction of a methyl or methoxy at the 7 and 8-positions (compounds **12**–**16**) improved ligand binding with the dimethyl-substituted **15** (IC_50_ = 27 μM) being 10-fold more potent than the initial fragment hits. Cyclization of positions 7 and 8 was well tolerated with **17**–**20** all showing good inhibitory activity. However, **17** was shown to be auto florescent interfering with the biochemical assay at higher concentrations making accurate IC_50_ calculations a challenge. Crystal structures obtained of compounds **11** and **17** (Figure 4) showed that the fragment was bound at the S4 pocket, inducing a conformational change in the highly flexible BL2 loop region to form a pi stacking interaction with Tyr-268 and a hydrogen bond with Asp-164. Based on the ligand binding pose there were two clear vectors for further expansion. Fragments could be grown from the piperidine nitrogen to access the S3 pocket of the binding site and block substrate access to the glycine channel. Alternatively, introduction of larger substitutions at the 7-position could expand into the BL2 groove on the left-hand side of our molecules. Analysis of X-ray structures showed that cyclization caused a shift in the position of the fragment core to sit higher in the binding pocket, altering the left-hand exit vector to access to the BL2 groove. The altered expansion vector along with autofluorescence in the enzymatic inhibition assay led to compound **15** being selected for further development in preference to **17** despite the improved binding affinity.

**Table 1:**
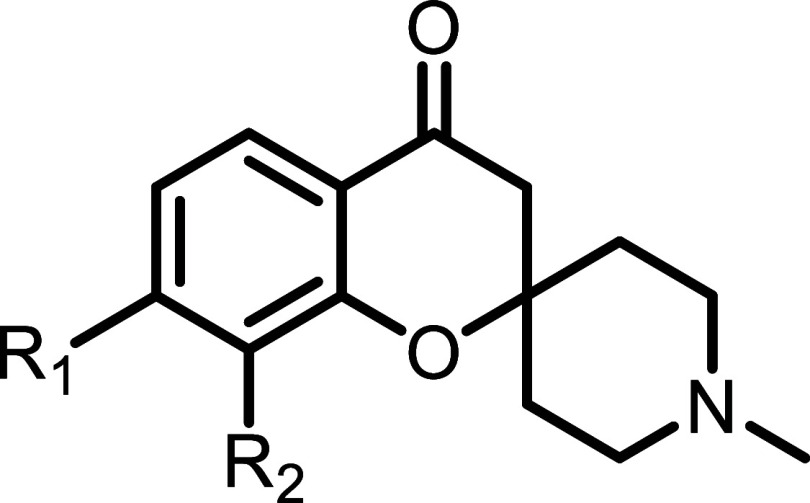

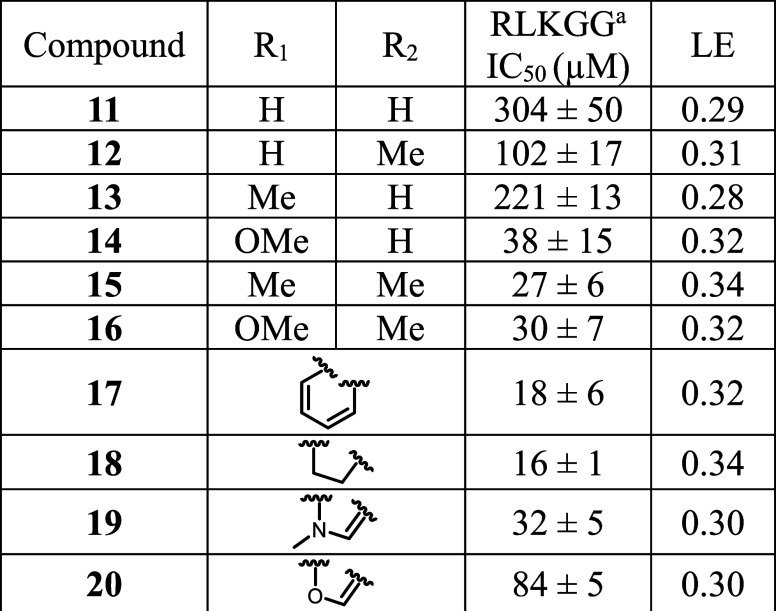
Enzyme Inhibition and LE Data for Spiro Chromanone Analogues **11**–**20**

a RLKGG biochemical IC_50_ represents the average of a minimum of two replicates ± standard deviation.

**Figure 4:**
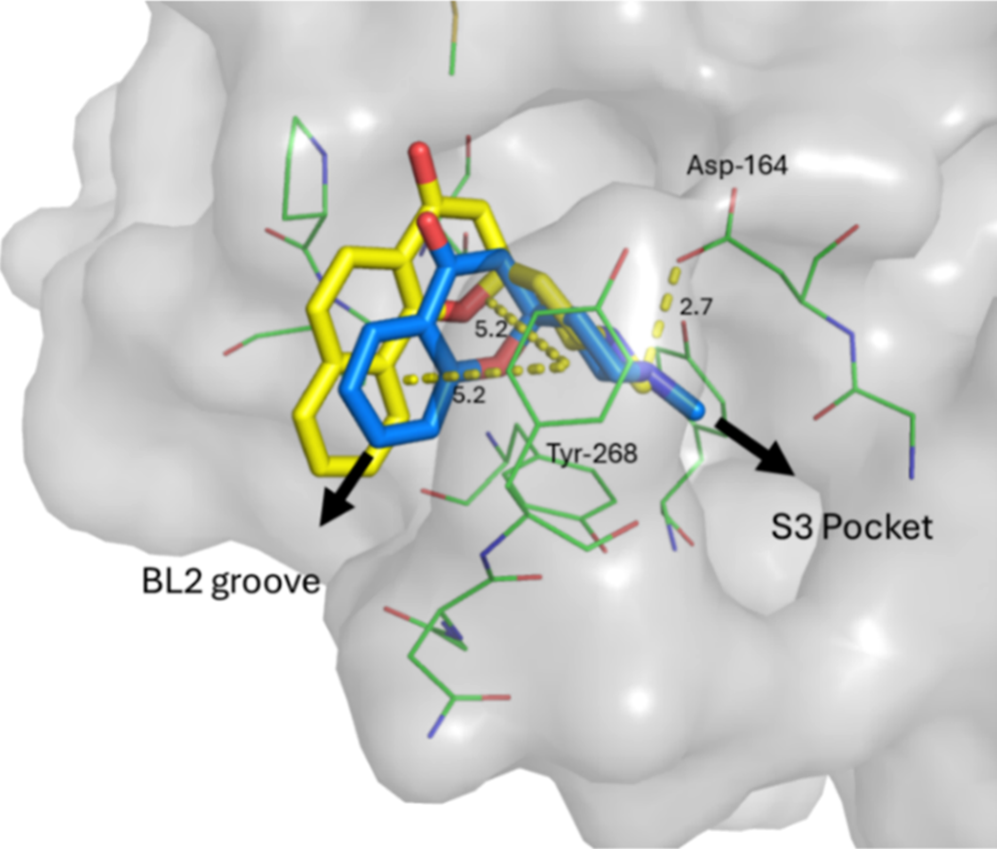
Overlay of crystal structures of fragment hit **11** (blue, PDB 9PUH) and analogue **17** (yellow, PDB 9PUJ) shown as sticks bound to SARS-CoV-2 PL^Pro^. Surface is shown in gray and nearby residues as green lines with key interactions shown as yellow dashes. Potential fragment expansion vectors are highlighted with black arrows.

### Accessing the S3 Pocket and BL2 Groove

Due to the fragment binding pose and narrow binding site there was insufficient room to attach rings directly to the piperidine nitrogen in the core. Therefore, we attempted to access the S3 pocket through the introduction of flexible linkers (Table 2, compounds **21**–**25**). All linkers containing amines and unsubstituted alcohols resulted in a loss of activity; however, ethers were tolerated with a slight increase in binding affinity (compound **25**). The S3 pocket proved to be flexible enough to accommodate larger compounds such as **25** without losing affinity. It was deemed that a single methylene linker was sufficient to gain access to the S3 pocket while minimizing conformation flexibility, allowing for substituted ring systems to have a fixed vector for further elaboration. Although various ring systems were tolerated, inclusion of a nitrogen at the 2-position proved to be essential, improving binding affinity by more than 10-fold compared to the phenyl (compounds **26** and **27**). Interestingly position of the nitrogen was found to be highly selective with compound **28** being greater than 20-fold less potent than **27**. Substitution of the pyridine at the 2 and 3-positions showed a slight increase in affinity, highlighting vectors for further expansion (compounds **29** and **30**). Extension off the fragment core at the 7-position was well tolerated with multiple rings and substitutions able to occupy the BL2 groove and provide a modest increase in activity (compounds **32**–**43**). The relatively broad tolerance to substitution of this series suggests that substitutions that access the BL2 groove may be used in the future to modulate the physiochemical properties of our lead compounds. Of the ring systems that we examined, the methylpyridine and methyl pyrazole showed the best activity with compound **37** being selected for further elaboration.

**Table 2:**
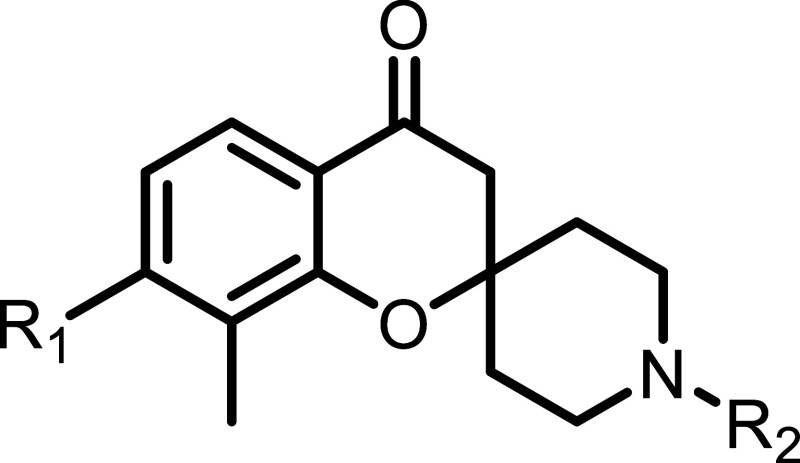

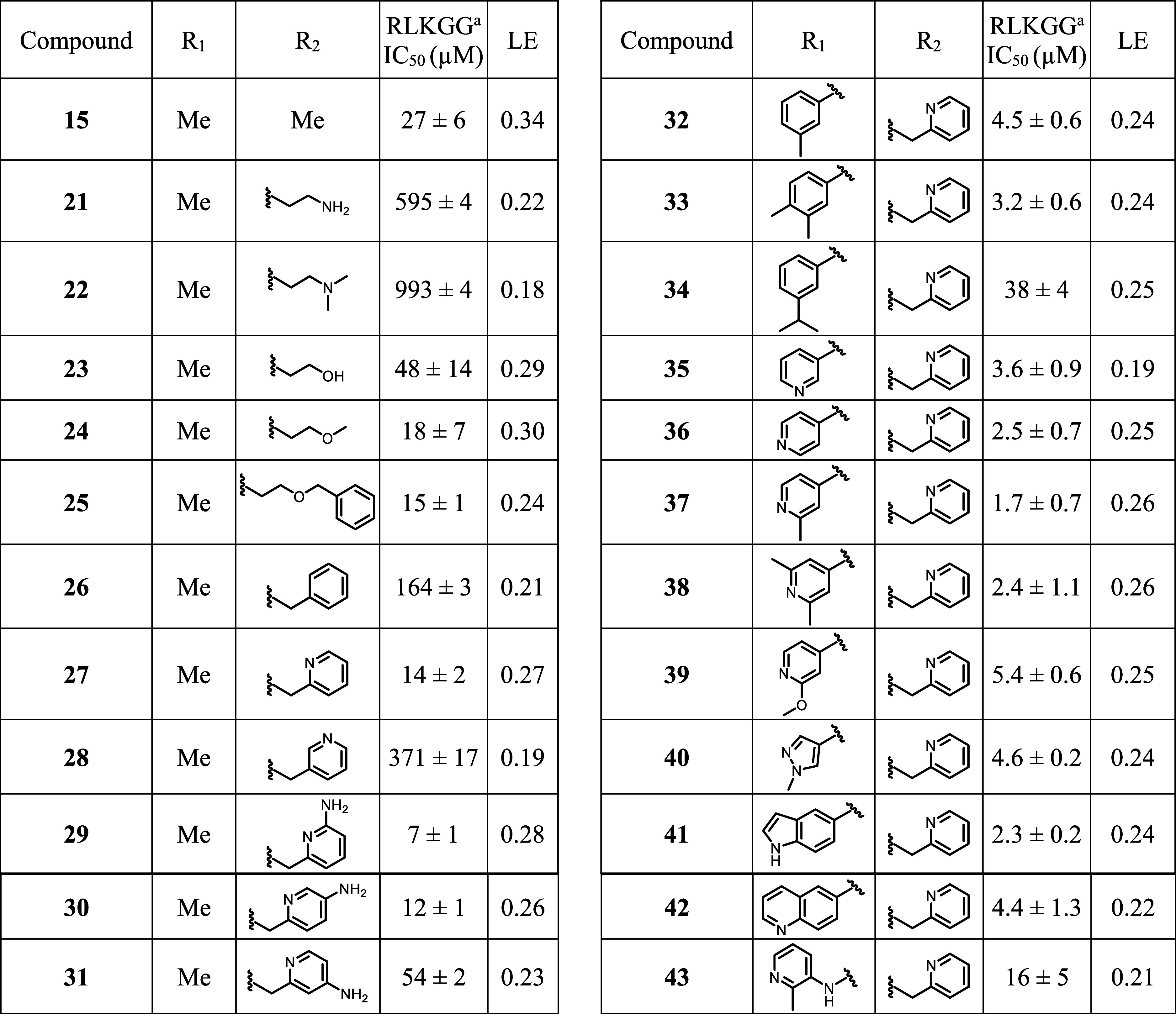
Enzyme Inhibition and LE Data for Elaborated Fragments **21**–**43**

a RLKGG biochemical IC_50_ represents the average of a minimum of two replicates ± standard deviation.

Crystal structures of **27** and **37** bound to PL^Pro^ showed that the position and binding mode of the fragment core was mostly maintained, forming a hydrogen bond with Asp-164 and a pi stacking interactions with Tyr-268 in the BL2 loop (Figure 5). The introduction of the nitrogen at the 2-position on the right-hand side pyridine formed an intramolecular hydrogen bond with the piperidine nitrogen of the fragment core. The methylpyridine in the BL2 groove was predominantly solvent exposed, explaining the tolerance for several heteroaromatic ring systems during the expansion of the left-hand side. Analysis of the binding pose highlighted two avenues for further compound development. Expansion off the 3-position of the right-hand side pyridine as seen in **29** could occupy a subsite at the top of the S3 pocket and potentially form interactions with Glu-167 and Lys-157. Alternatively, introduction of a flexible linker at the 5-position could gain access to the glycine channel, allowing for the development of irreversible inhibitors.

**Figure 5:**
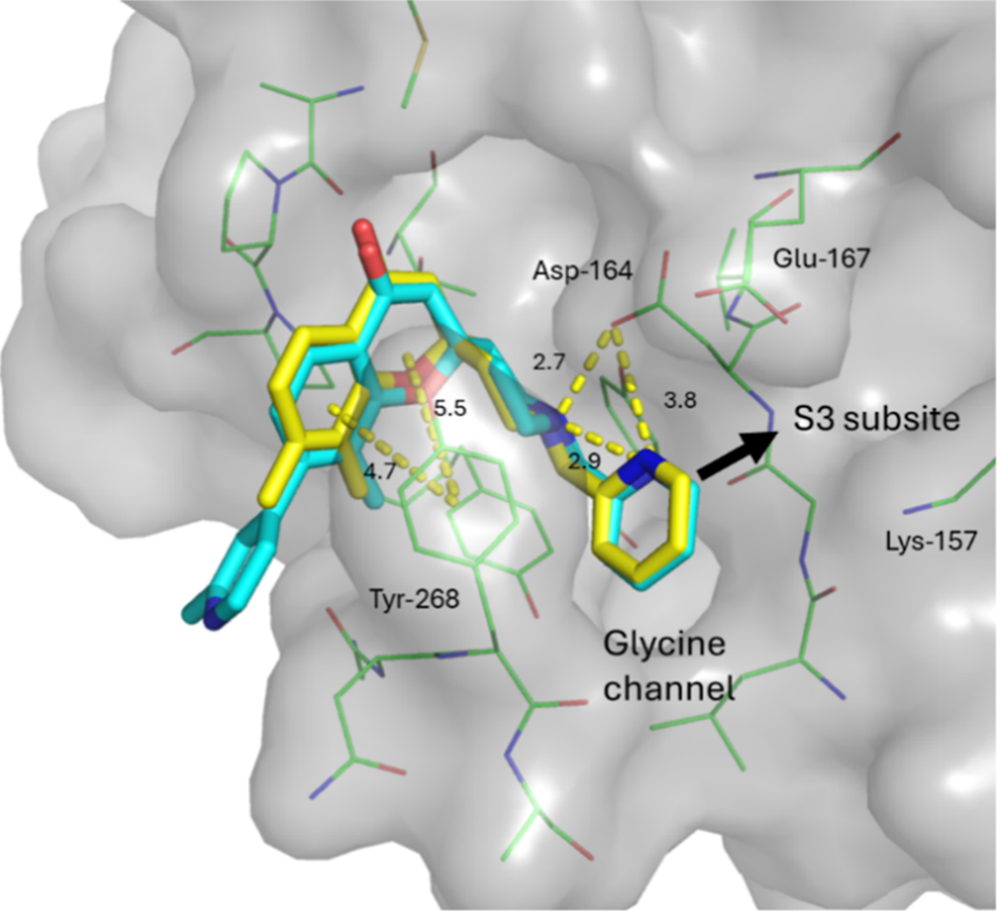
Overlay of crystal structures of compounds **27** (yellow, PDB 9PUY) and **37** (teal, PDB 9PV6) shown as sticks bound to SARS-CoV-2 PL^Pro^. Surface is shown in gray and nearby residues as green lines with key interactions shown as yellow dashes. Potential fragment expansion vectors are highlighted with black arrows.

### Expansion into the S3 Subsite

Although removal of the carbonyl from the spiro chromanone core (compound **44**) resulted in a slight decrease in binding affinity, it was found to be beneficial to cellular activity, as a result subsequent elaboration of compound **37** focused on the des-carbonyl core analogues (Table 3). Attempts to access the S3 subsite via expansion at the 2-position proved to be well tolerated with multiple aromatic and aliphatic rings providing an increase in binding affinity (compounds **45**–**52**). While both pyridine and pyrazole analogues had increased activity it was the basic amine containing methyl piperazine (compound **46**) that was most potent, with an IC_50_ of 0.4 μM and a 9-fold improvement compared to **44**. Inclusion of a basic amine proved to be essential for binding activity, with analogues that either reduced basicity (compound **50**) or removed the tertiary amine (compounds **51** and **52**) seeing a 3–10 fold decrease in activity. Furthermore, introduction of a chiral methyl (compound **49**) into the piperazine ring appeared to have minimal effect on overall binding activity.

**Table 3:**
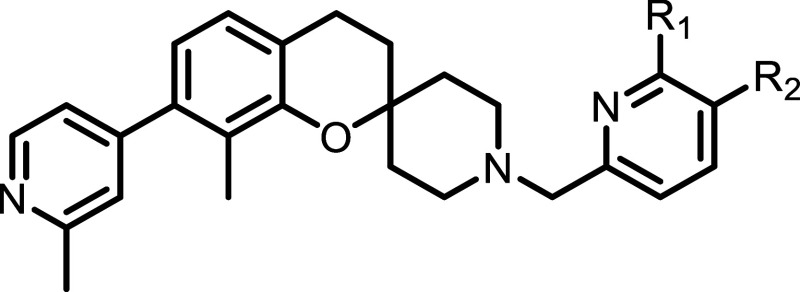

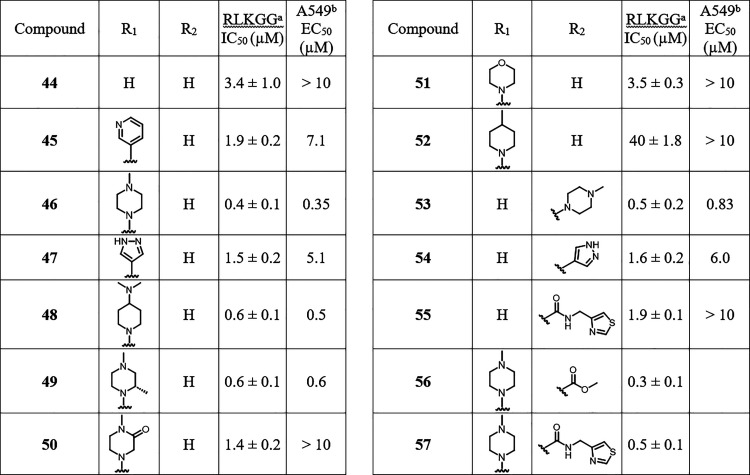
Enzyme Inhibition and Cellular Activity Data for Lead Compounds **44**–**57**

a RLKGG biochemical IC_50_ represent the average of a minimum of four replicates ± standard deviation.

b A549 cellular EC_50_ represent the average of three replicates.

A similar SAR trend was observed for substitutions at the 3-position (compounds **53** and **54**) with the methyl piperazine containing analogue **53** proving the most active (IC_50_ = 0.5 μM) in the series. Disubstitution of the pyridine or pyrazole at both the 2 and 3-positions was trialled but proved too bulky for the reaction to go to completion. It was proposed that introduction of an amide or ester at the 3-position (compounds **55**–**57**) could form interactions with nearby Lys-157. However, the inclusion in both mono and disubstituted systems failed to give any meaningful increase in binding affinity. Crystal structures of **46** and its analogues obtained by competitive soaking confirmed that substitutions at the 2 and 3-positions accessed the top of the S3 pocket with the basic amines forming a hydrogen bond with Glu-167 (Figure 6).

**Figure 6:**
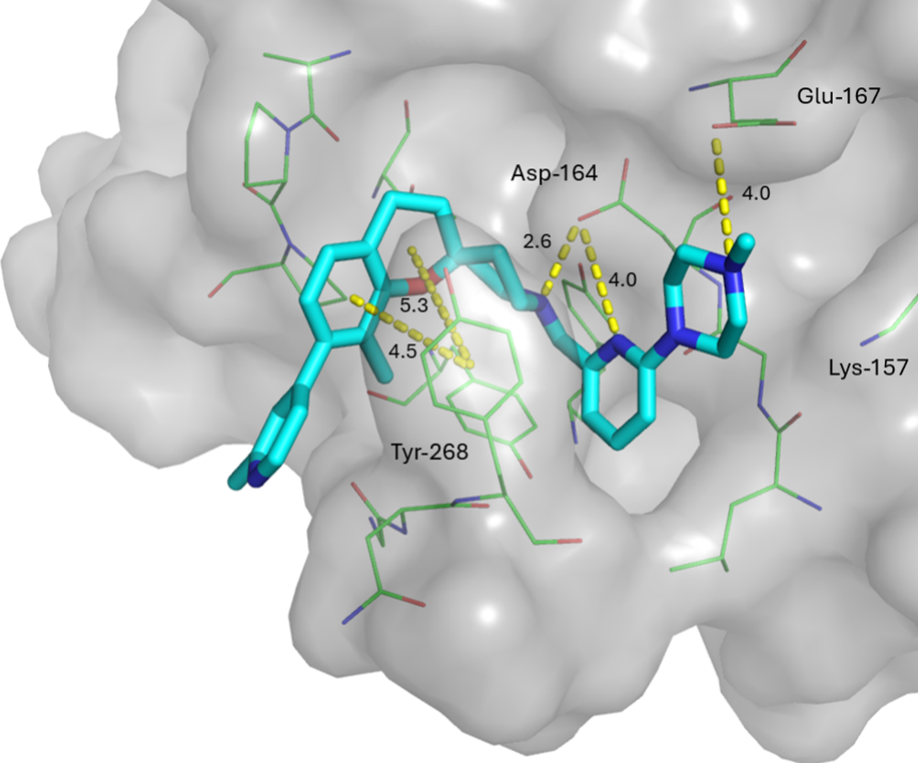
Crystal structure of **46** (PDB 9PV9) shown as teal sticks bound to SARS-CoV-2 PL^Pro^. Surface is shown in gray and nearby residues as green lines with key interactions shown as yellow dashes.

### Cellular Antiviral Activity and Resistance Development

Initial testing in SARS-CoV-2 infected A549 lung cells showed that most of the tested compounds exhibited antiviral activity, with several compounds exhibiting submicromolar inhibitory activity. Compound **46** was found to be the most potent, with a cellular activity comparable to its IC_50_. Substitution position seemed to have minimal effect on cellular activity, with 3-position analogues **53** and **54** exhibiting similar activity and SAR trends to compounds **46** and **47**. Inclusion of a basic amine was crucial for cellular activity and reduction of basicity through substitution with a less basic heterocycle or introduction of an electron withdrawing group (compounds **47** and **50**) resulted in a greater than 10-fold reduction in cell activity. Additionally, substitution of the piperazine for a morpholine or nonbasic piperidine (compounds **51** and **52**) showed no sign of cellular activity even at the highest tested concentration of 20 μM.

To examine the resistance development that may occur with this class of inhibitors, passaging studies were conducted with compound **46**. Six lineages were incubated with increasing amounts of compound **46** to a final concentration of 7 μM (Figure 7A). Higher ligand and DMSO concentrations were trialled but resulted in a loss of cell viability. Compound **46** showed no signs of drug-resistant mutations occurring, with a less than 1-fold reduction in EC_50_ between passages 0 and 8 (Figure 7B). In addition, sequencing of the 6 lineages showed no signs of recurring mutations in PL^Pro^ across multiple cell lines, one lineage did display a Q157R mutation but the distance from the binding site meant there was no effect on ligand binding and inhibition (Figure 7C). A T35K mutation was frequently observed in NSP-16 across multiple lineages, but we are currently unsure as to why this was observed or its biological significance. The good correlation between the IC_50_ and cellular activity, paired with its high tolerance to mutation in passaging studies suggests that compound **46** is a promising target for further optimization.

**Figure 7:**
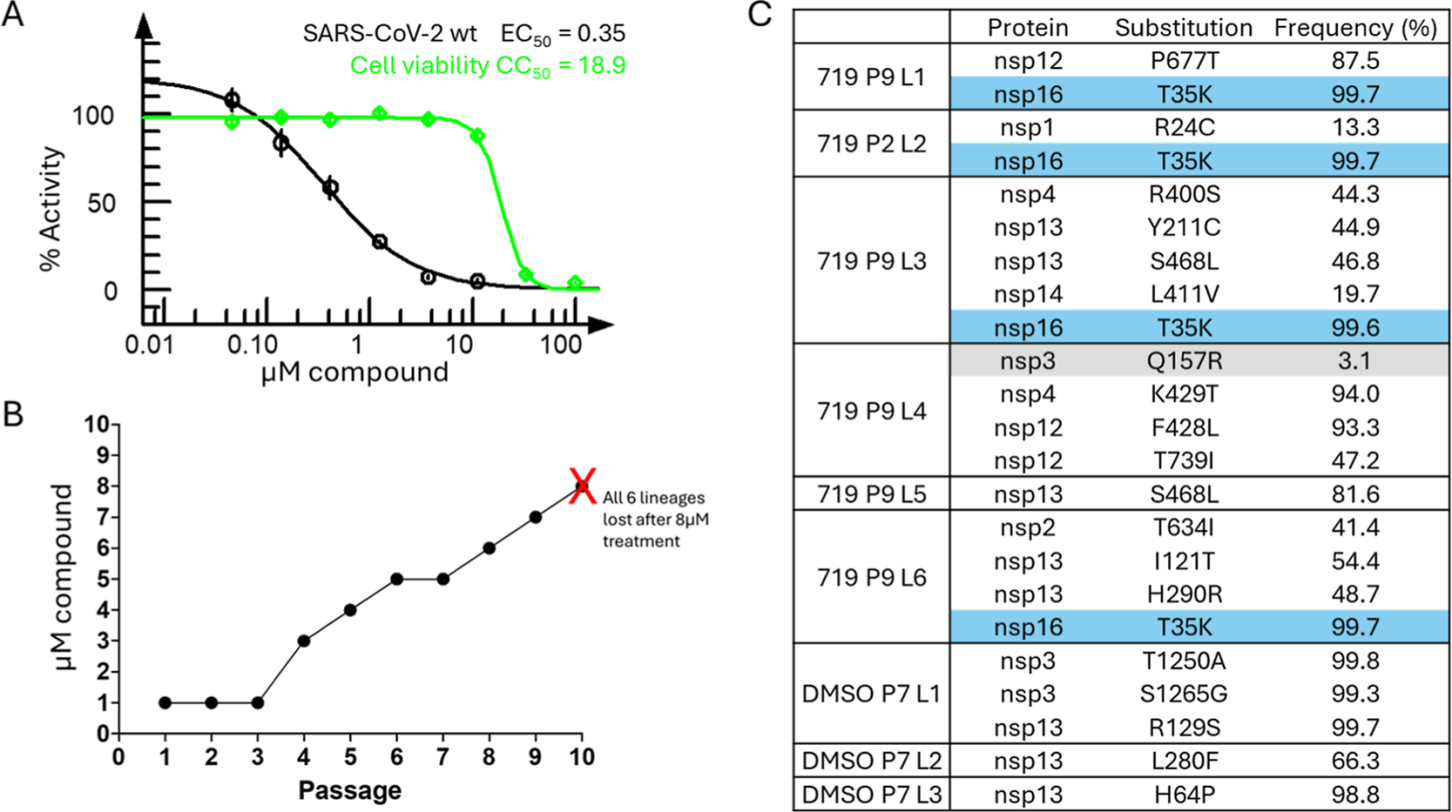
Cellular and resistance profiling of compound 46. (A) SARS-CoV-2 antiviral activity and cytotoxicity of compound **46** in A549 cells shown in black and green, respectively. Data points represent the average of ≥ 2 replicates, and error bars represent the standard deviation. (B) Dose escalation for passaging studies with no cell loss and cytotoxicity until passage 10 grown in 8 μM of compound **46**. (C) Identified amino acid mutations in SARS-CoV-2 with their frequency. Most frequent mutation observed in multiple cell lines highlighted in blue and mutations in PL^Pro^ highlighted in gray.

### Chemistry

The synthesis of the spiro-chromanone core intermediates **I**-**9** and **I**-**10** which were used for subsequent derivatization is detailed in Scheme 1. Acylation of a 2,3-disubstituted phenol (**I**-**1** and **I**-**2**) followed by Fries rearrangement with aluminum trichloride was used to generate hydroxy-acetophenones **I**-**5** and **I**-**6**. Cyclization with *N*-Boc-piperidin-4-one and pyrrolidine in methanol was used to generate the chromanone cores **I**-**7** and **I**-**8**. A Boc deprotection of chromanone **I**-**7** and **I**-**8** using TFA in DCM was used to afford key intermediates **I**-**9** and **I**-**10**. Chromanone **I**-**9** was used to generate analogues **21**–**31** via either a nucleophilic substitution with alkyl halides and potassium carbonate in acetone or a reductive amination with an aryl aldehyde, acetic acid and sodium triacetoxyborohydride in DCM. Key intermediate **I**-**11** was afforded through the reductive amination of chromanone **I**-**10** and picolinaldehyde in DCM. Intermediate **I**-**11** underwent a Suzuki reaction with various boronic acid/esters to generate analogues **32**–**42** and a Buchwald coupling for compound **43**.

**Scheme 1:**
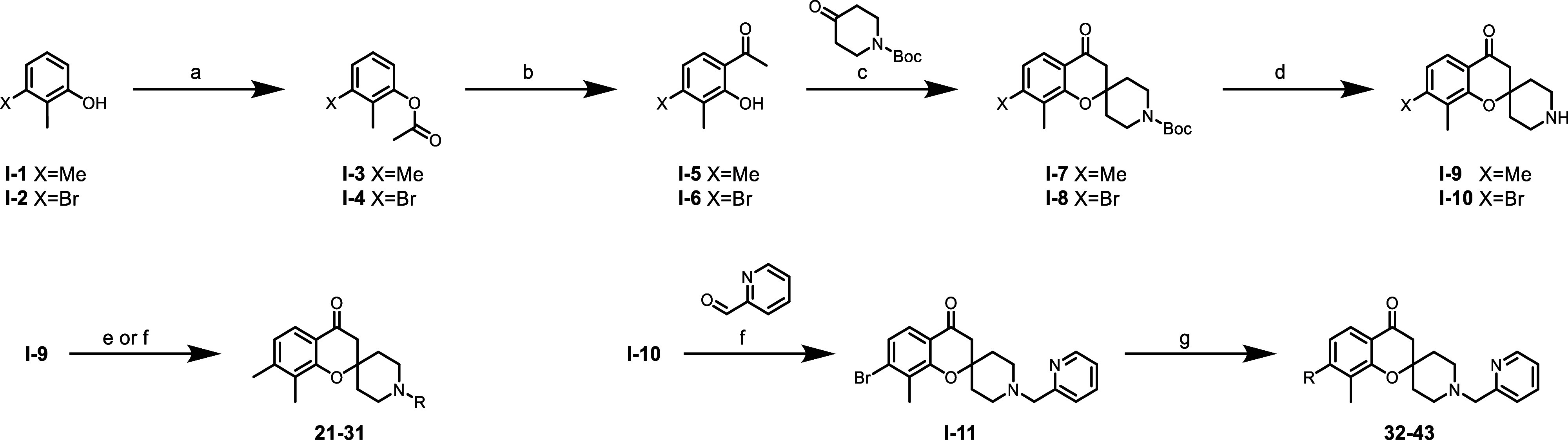
Synthesis of Elaborated Fragments **21**–**43** ^a^

a Reagents and conditions. (a) Acetyl chloride, Et_3_N, DCM, rt; (b) AlCl_3_, sealed tube, 135 °C; (c) pyrrolidine, MeOH, 80 °C; (d) TFA, DCM, 0 to 30 °C; (e) alkyl halide, K_2_CO_3_, acetone, 60 °C; (f) aldehyde, STAB, acetic acid, DCM, 55 °C; (g) boronic acid/esters, Pd­(dppf)­Cl_2_·DCM, Cs_2_CO_3_, dioxane, water, 90 °C.

The synthesis of spiro-chromane analogues **44**–**57** is detailed in Scheme 2. Key chromanone intermediate **I**-**12** underwent a sodium borohydride reduction followed by alcohol elimination using triethyl silane in TFA to afford key intermediate **I**-**14**. Spiro-chromane **I**-**14** was used in a reductive amination with various halo and ester substituted pyridine-2-aldehydes to afford intermediates **I**-**15** to **I**-**18**. Analogues **44**–**54** were generated through a Suzuki or Buchwald coupling with various boronic acids and secondary amines, respectively. To generate amides **55** and **58**, their respective intermediates **I-17** and **I**-**18** underwent a lithium hydroxide hydrolysis followed by a HATU mediated amide formation.

**Scheme 2:**
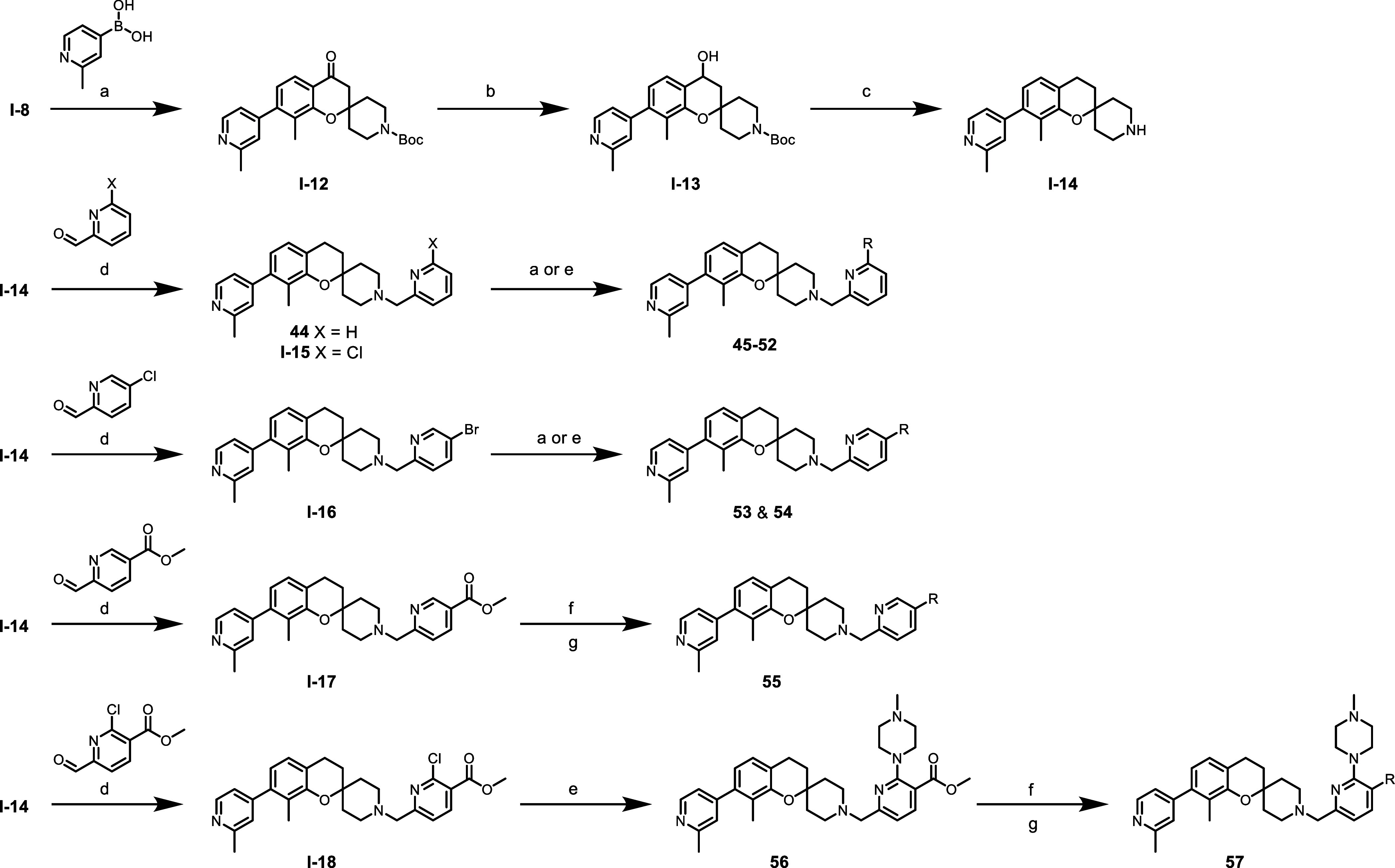
Synthesis of S3 Subsite Binders **44**–**57** ^a^

a Reagents and conditions. (a) Aryl boronic acid, Pd­(dppf)­Cl_2_·DCM, Cs_2_CO_3_, dioxane, water, 90 °C; (b) NaBH_4_, MeOH, 0 to rt; (c) Et_3_SiH, TFA, 80 °C; (d) aryl aldehyde, STAB, acetic acid, DCM, 55 °C; (e) secondary amine, Pd_2_(dba)_3_, Cs_2_CO_3_, RuPhos, dioxane, 100 °C; (f) LiOH, MeOH, water, 40 °C; (g) substituted amine, HATU, DIPEA, DMF, rt.

## Conclusions

Here we have described the discovery of a novel class of PL^Pro^ inhibitors using fragment-based methods and structure-based design. We identified several chemical scaffolds (including spiro chromanone fragments) that bound at the S4 pocket with an IC_50_∼500 μM. X-ray structures of cocomplexes were used to guide the optimization of fragments, achieving a ∼1000-fold improvement in activity. Fragments were grown to occupy the BL2 groove and S3 pocket while maintaining the initial fragment binding mode. The addition of basic amines at the S3 subsite led to elaborated compounds with submicromolar potency. Initial profiling of cellular **46** shows encouraging submicromolar antiviral cellular activity and resistance profiles. Notably, this is the first example of PL^Pro^ inhibitors with cellular activity that are not based on the structure of the GRL-0617 scaffold. These compounds represent a promising starting point for the development of a novel class of SARS-CoV-2 PL^Pro^ inhibitors.

## Experimental Section

### General Chemistry

All chemical reagents and reaction solvents were purchased from commercial suppliers and used as received. NMR spectra were recorded on a Bruker AVIII-HD 400 spectrometer and processed using Mestrenova 15.0.1. Chemical shifts are reported in parts per million (ppm) relative to residual nondeuterated solvent signals, CDCl_3_ (^1^H, 7.26 ppm), DMSO-*d*_6_ (^1^H, 2.50 ppm), and CD_3_OD (^1^H, 3.31 ppm), and coupling constants are reported in hertz (Hz). The following abbreviations (or a combination, thereof) are used to describe splitting patterns: s, singlet; d, doublet; t, triplet; q, quartet; p, pentet; m, multiplet; br, broad. All final compounds were of >95% purity as measured by analytical reversed-phase HPLC. Analytical HPLC was performed on an Agilent 1200 series system with UV detection at 214 and 254 nm, along with evaporative light scattering detection (ELSD). Low-resolution mass spectra were obtained on an Agilent 6140 mass spectrometer with electrospray ionization (ESI). LCMS experiments were performed with the following parameters: Phenomenex Kinetex 2.6 μm XB-C18 100 Å LC column (50 mm × 2.1 mm); 2 min gradient, 5–95% MeCN in H_2_O, and 0.1% TFA. Silica gel chromatography was performed using a Teledyne ISCO Combiflash R_f_ system, eluting with varying concentrations of EtOAc in hexanes or MeOH in CH_2_Cl_2_. Preparative reversed-phase HPLC was performed on a Gilson HPLC equipped with a Phenomenex Kinetex C18 column, using varying concentrations of MeCN in H_2_O, and 0.1% TFA. Solvents for reactions, extraction, and washing were ACS grade, and solvents for chromatography were HPLC grade.

#### 2,3-Dimethylphenyl Acetate (**I**-**3**)

2,3-Dimethylphenol (5.08 g, 1.0 eq, 41.6 mmol) was dissolved in DCM (100 mL) and cooled to 0 °C in an ice bath. Triethylamine (8.42 g, 2.0 eq, 83.2 mmol) and acetyl chloride (4.90 g, 1.5 eq, 62.4 mmol) were added and the reaction was stirred for 5 min before being warmed to room temp and stirred for a further 3 h. At completion the reaction was diluted with water (150 mL) and extracted with DCM (3 × 50 mL). The organic layers were pooled, washed with brine, dried over anhydrous magnesium sulfate and concentrated in vacuo to give crude product. Further purification was conducted by flash column chromatography with a gradient of 0–30% EtOAc in hexanes to give product (6.2 g, 91%) as a clear oil. LCMS (*m*/*z*) 165.3 (M + H)^+^ *t*_*R*_ = 1.73 min.

#### 1-(2-Hydroxy-3,4-dimethylphenyl)­ethan-1-one (**I**-**5**)

2,3-Dimethylphenyl acetate **I**-**3** (6.20 g, 1.0 eq, 37.6 mmol) was placed in a sealed tube, aluminum trichloride (10.1 g, 2.0 eq, 75.4 mmol) was added and the reaction was heated to 135 °C for 2 h. At completion the reaction was cooled to room temp, dissolved in EtOAc (100 mL) and washed with water (2 × 50 mL) and brine (50 mL). The organic phase was dried over anhydrous magnesium sulfate and concentrated in vacuo to give crude product. Further purification was conducted by flash column chromatography with a gradient of 0–20% EtOAc in hexanes to give product (5.5 g, 89%) as a yellow oil. LCMS (*m*/*z*) 165.3 (M + H)^+^ *t*_*R*_ = 1.84 min.

#### *Tert*-Butyl 7,8-dimethyl-4-oxospiro­[chromane-2,4′-piperidine]-1′-carboxylate (**I**-**7**)

1-(2-Hydroxy-3,4-dimethylphenyl)­ethan-1-one **I**-**5** (5.50 g, 1.0 eq, 33.4 mmol) and *tert*-butyl 4-oxopiperidine-1-carboxylate (7.31 g, 1.1 eq, 36.7 mmol) were dissolved in MeOH (75 mL) and the reaction was cooled to 0 °C. Pyrrolidine (3.70 g, 1.5 eq, 50.1 mmol) was added and the reaction was stirred for 5 min before being heated to 80 °C and left to react for 3 h. At completion the reaction was cooled to room temp, diluted with EtOAc (150 mL) and washed with water (2 × 50 mL) and brine (50 mL), the organic phase was then dried over anhydrous magnesium sulfate and concentered in vacuo to give crude product. Further purification was conducted by flash column chromatography with a gradient of 0–20% EtOAc in hexanes to give product (11.4 g, 99%) as an off-white powder. ^1^H NMR (CDCl_3_): δ 7.62 (d, *J* = 8.0 Hz, 1H), 6.82 (d, *J* = 8.0 Hz, 1H), 3.92 (br s, 2H), 3.16 (t, *J* = 12.9 Hz, 2H), 2.67 (s, 2H), 2.30 (s, 3H), 2.19 (s, 3H), 2.01 (br s, 2H), 1.59 (ddd, *J* = 13.9, 12.3, 4.9 Hz, 2H), 1.46 (s, 9H). LCMS (*m*/*z*) 290.2 (M + H-56­(*t*-butyl))^+^ *t*_*R*_ = 2.13 min.

#### 7,8-Dimethylspiro­[chromane-2,4′-piperidin]-4-one (**I**-**9**)

*Tert*-butyl 7,8-dimethyl-4-oxospiro-[chromane-2,4′-piperidine]-1′-carboxylate **I**-**7** (1.01 g, 1.0 eq, 2.91 mmol) was dissolved in DCM (30 mL) and the solution was cooled to 0 °C. TFA (3.30 g, 10.0 eq, 29.1 mmol) was added dropwise and the reaction was left stirring for 10 min before warming to room temp and reacting for another 2 h. At completion the reaction was cooled to 0 °C and quenched by the dropwise addition of NaOH (aq, 2M) until a pH of 9 was reached. The reaction was diluted with water (50 mL) and extracted with DCM (2 × 20 mL) and EtOAc (20 mL). The organic layers were pooled, dried over anhydrous magnesium sulfate and concentrated in vacuo to give product (700.1 mg, 98%) as an orange solid which was carried on without further purification. ^1^H NMR (MeOD) δ 7.53 (d, *J* = 8.0 Hz, 1H), 6.83 (d, *J* = 8.1 Hz, 1H), 3.07 (td, *J* = 12.4, 2.8 Hz, 2H), 3.03–2.92 (m, 2H), 2.30 (s, 3H), 2.23 (s, 3H), 2.10–2.00 (m, 2H), 1.78–1.65 (m, 2H). LCMS (*m*/*z*) 246.3 (M + H)^+^ *t*_*R*_ = 1.30 min.

#### 3-Bromo-2-methylphenyl Acetate (I-4)

Prepared as described for **I**-**3**, clear oil 21.2 g, 92%. ^1^H NMR (CDCl_3_) δ 7.45 (dd, *J* = 8.0, 1.3 Hz, 1H), 7.07 (t, *J* = 8.0 Hz, 1H), 6.99 (dd, *J* = 8.0, 1.3 Hz, 1H), 2.33 (s, 3H), 2.26 (s, 3H). LCMS (*m*/*z*) 229.0 (M + H)^+^ *t*_*R*_ = 1.79 min.

#### 1-(4-Bromo-2-hydroxy-3-methylphenyl)­ethan-1-one (**I**-**6**)

Prepared as described for **I**-**5**, off white crystals 14.8 g, 73.7%. ^1^H NMR (CDCl_3_): δ 7.43 (d, *J* = 8.6 Hz, 1H), 7.10 (d, *J* = 8.6 Hz, 1H), 2.61 (s, 3H), 2.35 (s, 3H). LCMS (*m*/*z*) 229.0 (M + H)^+^ *t*_*R*_ = 1.93 min.

#### *Tert*-Butyl 7-bromo-8-methyl-4-oxospiro­[chromane-2,4′-piperidine]-1′-carboxylate (I-8)

Prepared as described for **I**-**7**, off white powder 16.2 g, 91%. ^1^H NMR (CDCl_3_): δ 7.58 (d, *J* = 8.5 Hz, 1H), 7.22 (d, *J* = 8.5 Hz, 1H), 3.95 (br d, *J* = 13.5 Hz, 2H), 3.15 (t, *J* = 13.6 Hz, 2H), 2.70 (s, 2H), 2.37 (s, 3H), 2.02 (dd, *J* = 13.6, 1.3 Hz, 2H), 1.63 (dd, *J* = 12.5, 4.9 Hz, 2H), 1.46 (s, 9H). LCMS (*m*/*z*) 354.1 (M + H-56­(*t*-butyl))^+^ *t*_*R*_ = 2.23 min.

#### 7-Bromo-8-methylspiro­[chromane-2,4′-piperidin]-4-one (**I**-**10**)

Prepared as described for **I**-**9**, clear oil 23.0 g, 94%. LCMS (*m*/*z*) 229.0 (M + H)^+^ *t*_*R*_ = 1.79 min.

#### 1′-(2-(Dimethylamino)­ethyl)-7,8-dimethylspiro­[chromane-2,4′-piperidin]-4-one (**22**)

Intermediate **I**-**9** (51.2 mg, 1.0 eq, 0.208 mmol), 2-bromo-*N*,*N*-dimethylethan-1-amine (38.3 mg, 1.2 eq, 0.251 mmol) and potassium carbonate (73.2 mg, 2.5 eq, 0.529 mmol) were dissolved in acetone (1 mL) and the reaction was heated to 60 °C for 4 h. At completion the reaction was diluted with water (20 mL), basified with NaOH (aq, 2M) and extracted with DCM (2 × 20 mL) and EtOAc (20 mL). The organic layers were pooled, dried over anhydrous magnesium sulfate and concentrated in vacuo to give product. Further purification was conducted via reverse phase preparative HPLC with a gradient of 5–60% MeCN in water to give product as a yellow powder 47.6 mg 72%. ^1^H NMR (CDCl_3_): δ 7.63 (d, *J* = 8.1 Hz, 1H), 6.83 (d, *J* = 8.0 Hz, 1H), 3.13 (s, 2H), 3.03 (d, *J* = 5.9 Hz, 2H), 2.91 (s, 2H), 2.79 (s, 6H), 2.69 (s, 2H), 2.31 (s, 3H), 2.19 (s, 3H), 2.17–1.88 (m, 6H). LCMS (*m*/*z*) 317.2 (M + H)^+^ *t*_*R*_ = 1.26 min.

#### 1′-(2-Aminoethyl)-7,8-dimethylspiro­[chromane-2,4′-piperidin]-4-one (**21**)

Prepared as described for **22**, yellow powder 34.8 mg. ^1^H NMR (MeOD): δ 7.60 (d, *J* = 8.0 Hz, 1H), 6.92 (d, *J* = 8.1 Hz, 1H), 3.60 (br s, 2H), 3.56–3.42 (m, 4H), 2.84 (s, 2H), 2.39 (s, 2H), 2.34 (s, 3H), 2.29 (s, 3H), 2.27–2.15 (m, 4H). LCMS (*m*/*z*) 289.3 (M + H)^+^ *t*_*R*_ = 1.21 min.

#### 1′-(2-Hydroxyethyl)-7,8-dimethylspiro­[chromane-2,4′-piperidin]-4-one (**23**)

Prepared as described for **22**, yellow powder 24.1 mg. ^1^H NMR (CDCl_3_): δ 7.67 (d, *J* = 8.0 Hz, 1H), 6.89 (d, *J* = 8.0 Hz, 1H), 4.10–4.03 (m, 2H), 3.58 (br d, *J* = 12.1 Hz, 2H), 3.23–3.11 (m, 4H), 2.79 (s, 2H), 2.57 (t, *J* = 13.4 Hz, 2H), 2.32 (s, 3H), 2.27 (d, *J* = 13.9 Hz, 2H), 2.20 (s, 3H). LCMS (*m*/*z*) 290.2 (M + H)^+^ *t*_*R*_ = 1.31 min.

#### 1′-(2-Methoxyethyl)-7,8-dimethylspiro­[chromane-2,4′-piperidin]-4-one (**24**)

Prepared as described for **22**, white powder 47.9 mg. ^1^H NMR (CDCl_3_): δ 7.63 (d, *J* = 8.0 Hz, 1H), 6.81 (d, *J* = 8.0 Hz, 1H), 3.59 (s, 2H), 3.36 (s, 3H), 2.85 (s, 2H), 2.68 (s, 2H), 2.30 (s, 3H), 2.20 (s, 3H), 2.08 (d, *J* = 13.6 Hz, 2H), 1.91 (br s, 2H), 1.61 (br s, 4H). LCMS (*m*/*z*) 304.2 (M + H)^+^ *t*_*R*_ = 1.39 min.

#### 1′-(2-(Benzyloxy)­ethyl)-7,8-dimethylspiro­[chromane-2,4′-piperidin]-4-one (**25**)

Prepared as described for **22**, yellow powder 63.1 mg. ^1^H NMR (CDCl_3_): δ 7.55 (d, *J* = 8.0 Hz, 1H), 7.31–7.17 (m, 5H), 6.73 (d, *J* = 8.0 Hz, 1H), 4.47 (s, 2H), 3.56 (t, *J* = 5.6 Hz, 2H), 2.69 (d, *J* = 11.0 Hz, 2H), 2.63 (t, *J* = 5.3 Hz, 2H), 2.60 (s, 2H), 2.44 (t, *J* = 12.5 Hz, 2H), 2.23 (s, 3H), 2.12 (s, 3H), 2.03–1.91 (m, 2H), 1.74 (t, *J* = 12.2 Hz, 2H). LCMS (*m*/*z*) 380.2 (M + H)^+^ *t*_*R*_ = 1.67 min.

#### 1′-Benzyl-7,8-dimethylspiro­[chromane-2,4′-piperidin]-4-one (**26**)

Intermediate **I**-**9** (76.6 mg, 1.0 eq, 0.312 mmol), benzaldehyde (39.8 mg, 1.2 eq, 0.375 mmol) and sodium triacetoxyhydroborate (206.6 mg, 3.1 eq, 0.975 mmol) were dissolved in DCM (1 mL), 3–4 drops of acetic acid was added and the reaction was heated to 55 °C for 3 h. At completion the reaction was diluted with water (10 mL), basified with the addition of sodium hydroxide (aq, 2 M) then extracted with DCM (2 × 10 mL) and EtOAc (10 mL). The organic layers were combined, dried over anhydrous magnesium sulfate and concentrated in vacuo to give crude product. Further purification was conducted via reverse phase preparative HPLC with a gradient of 5–60% MeCN in water to give product as a yellow solid 34.9 mg 33%. ^1^H NMR (CDCl_3_): δ 7.61 (d, *J* = 8.0 Hz, 1H), 7.40–7.27 (m, 5H), 6.80 (d, *J* = 8.0 Hz, 1H), 3.62 (br s, 2H), 2.71 (br s, 2H), 2.67 (s, 2H), 2.50 (br s, 2H), 2.30 (s, 3H), 2.15 (s, 3H), 2.05 (d, *J* = 12.8 Hz, 2H), 1.81 (br s, 2H). LCMS (*m*/*z*) 336.3 (M + H)^+^ *t*_*R*_ = 1.58 min.

#### 7,8-Dimethyl-1′-(pyridin-2-ylmethyl)­spiro­[chromane-2,4′-piperidin]-4-one (**27**)

Prepared as described for **26**, off white powder 82.4 mg. ^1^H NMR (CDCl_3_): δ 8.59 (d, *J* = 4.2 Hz, 1H), 7.69 (t, *J* = 6.9 Hz, 1H), 7.62 (d, *J* = 8.0 Hz, 1H), 7.49 (s, 1H), 7.22 (d, *J* = 6.2 Hz, 1H), 6.81 (d, *J* = 8.0 Hz, 1H), 3.88 (s, 2H), 2.99–2.72 (br m, 4H), 2.69 (s, 2H), 2.30 (s, 3H), 2.16 (s, 3H), 2.09 (d, *J* = 13.6 Hz, 2H), 1.94 (br s, 2H). LCMS (*m*/*z*) 337.2 (M + H)^+^ *t*_*R*_ = 1.44 min.

#### 7,8-Dimethyl-1′-(pyridin-3-ylmethyl)­spiro­[chromane-2,4′-piperidin]-4-one (**28**)

Prepared as described for **26**, yellow solid 25.4 mg, 24%. ^1^H NMR (CDCl_3_): δ 8.55 (d, *J* = 2.2 Hz, 1H), 8.52 (dd, *J* = 4.8, 1.7 Hz, 1H), 7.71 (d, *J* = 7.8 Hz, 1H), 7.61 (d, *J* = 8.0 Hz, 1H), 7.27 (dd, *J* = 7.8, 4.8 Hz, 1H), 6.80 (d, *J* = 8.0 Hz, 1H), 3.60 (s, 2H), 2.9 (br s, 2H), 2.67 (s, 2H), 2.49 (t, *J* = 11.5 Hz, 2H), 2.30 (s, 3H), 2.17 (s, 3H), 2.05 (dd, *J* = 14.5, 1.7 Hz, 2H), 1.77 (t, 10.5 Hz, 2H). LCMS (*m*/*z*) 337.3 (M + H)^+^ *t*_*R*_ = 1.27 min.

#### 1′-((6-Aminopyridin-2-yl)­methyl)-7,8-dimethylspiro­[chromane-2,4′-piperidin]-4-one (**29**)

Prepared as described for **26**, white powder 37.8 mg. ^1^H NMR (MeOD): δ 7.88 (dd, *J* = 8.9, 7.1 Hz, 1H), 7.59 (d, *J* = 8.0 Hz, 1H), 7.06 (d, *J* = 7.1 Hz, 1H), 7.02 (d, *J* = 9.0 Hz, 1H), 6.92 (d, *J* = 8.1 Hz, 1H), 4.50 (s, 2H), 3.54–3.36 (m, 4H), 2.84 (s, 2H), 2.39–2.32 (m, 2H), 2.35 (s, 3H), 2.29 (s, 3H), 2.20–2.09 (m, 2H). LCMS (*m*/*z*) 352.2 (M + H)^+^ *t*_*R*_ = 1.31 min.

#### 1′-((5-Aminopyridin-2-yl)­methyl)-7,8-dimethylspiro­[chromane-2,4′-piperidin]-4-one (**30**)

Prepared as described for **26**, yellow solid 29.2 mg. ^1^H NMR (MeOD): δ 8.09 (d, *J* = 2.8 Hz, 1H), 7.34 (d, *J* = 8.4 Hz, 1H), 7.16 (dd, *J* = 8.4, 2.8 Hz, 1H), 6.79 (d, *J* = 7.7 Hz, 1H), 6.66 (d, *J* = 7.7 Hz, 1H), 4.36 (s, 2H), 3.46–3.34 (m, 4H), 2.78 (t, *J* = 6.9 Hz, 2H), 2.20 (s, 3H), 2.09 (s, 3H), 1.95 (td, *J* = 13.9, 4.8 Hz, 2H), 1.86 (t, *J* = 6.9 Hz, 2H). LCMS (*m*/*z*) 352.2 (M + H)^+^ *t*_*R*_ = 1.47 min.

#### 1′-((4-Aminopyridin-2-yl)­methyl)-7,8-dimethylspiro­[chromane-2,4′-piperidin]-4-one (**31**)

Prepared as described for **26**, yellow powder 25.9 mg. ^1^H NMR (MeOD): δ 8.13 (d, *J* = 7.1 Hz, 1H), 7.59 (d, *J* = 8.0 Hz, 1H), 7.22 (d, *J* = 2.5 Hz, 1H), 6.95–6.87 (m, 2H), 4.56 (s, 2H), 3.57–3.36 (m, 4H), 2.84 (s, 2H), 2.38 (br s, 2H), 2.35 (s, 3H), 2.29 (s, 3H), 2.27–2.15 (m, 2H). LCMS (*m*/*z*) 352.3 (M + H)^+^ *t*_*R*_ = 1.25 min.

#### 7-Bromo-8-methyl-1′-(pyridin-2-ylmethyl)­spiro­[chromane-2,4′-piperidin]-4-one (**I**-**11**)

Prepared as described for **26**, using intermediate **I**-**10**, brown powder. ^1^H NMR (CDCl_3_): δ 8.61 (d, *J* = 4.5 Hz, 1H), 7.73 (dt, *J* = 7.8, 4.5 Hz, 1H), 7.57 (d, *J* = 8.5 Hz, 1H), 7.49 (d, *J* = 7.8 Hz, 1H), 7.28 (t, *J* = 8.0 Hz, 1H), 7.22 (d, *J* = 8.5 Hz, 1H), 4.00 (br s, 2H), 3.18–2.81 (m, 4H), 2.73 (s, 2H), 2.31 (s, 3H), 2.19–1.90 (m, 4H). LCMS (*m*/*z*) 401.1 (M + H)^+^ *t*_*R*_ = 1.51 min.

#### 8-Methyl-1′-(pyridin-2-ylmethyl)-7-(*m*-tolyl)­spiro­[chromane-2,4′-piperidin]-4-one (**32**)

Intermediate **I**-**11** (50.9 mg, 1.0 eq, 0.127 mmol), *m*-tolylboronic acid (21.5 mg, 1.25 eq, 0.158 mmol), cesium carbonate (123.8 mg, 3.0 eq, 0.380 mmol) and RuPhos (11.9 mg, 0.2 eq, 0.025 mmol) were dissolved in an 8:2 mixture of dioxane/water and purged with argon. Pd­(dppf)­Cl_2_·DCM (10.4 mg, 0.1 eq, 0.012 mmol) was added and the reaction was heated to 90 °C for 1 h. At completion the reaction was diluted with water, basified with NaOH (aq, 2M) and then extracted with DCM (2 × 10 mL) and EtOAc (10 mL). The organic layers were combined, dried over anhydrous magnesium sulfate and concentrated in vacuo to give crude product. Further purification was conducted via reverse phase preparative HPLC with a gradient of 5–70% MeCN in water to give product as a yellow solid 31.2 mg 60%. ^1^H NMR (CDCl_3_): δ 8.56 (d, *J* = 4.5 Hz, 1H), 7.73 (d, *J* = 8.1 Hz, 1H), 7.65 (td, *J* = 7.7, 1.8 Hz, 1H), 7.41 (d, *J* = 7.8 Hz, 1H), 7.32 (t, *J* = 7.5 Hz, 1H), 7.21–7.14 (m, 2H), 7.12–7.06 (m, 2H), 6.88 (d, *J* = 8.1 Hz, 1H), 3.73 (s, 2H), 2.78–2.68 (m, 4H), 2.58 (td, *J* = 11.7, 2.6 Hz, 2H), 2.41 (s, 3H), 2.17 (s, 3H), 2.10 (dd, *J* = 14.5, 2.7 Hz, 2H), 1.84 (td, *J* = 12.7, 5.0 Hz, 2H). LCMS (*m*/*z*) 413.3 (M + H)^+^ *t*_*R*_ = 1.75 min.

#### 7-(3,4-Dimethylphenyl)-8-methyl-1′-(pyridin-2-ylmethyl)­spiro­[chromane-2,4′-piperidin]-4-one (**33**)

Prepared as described for **32**, yellow solid. ^1^H NMR (CDCl_3_): δ 8.56 (d, *J* = 5.1 Hz, 1H), 7.72 (d, *J* = 8.1 Hz, 1H), 7.65 (t, *J* = 7.7 Hz, 1H), 7.40 (d, *J* = 7.8 Hz, 1H), 7.22–7.14 (m, 2H), 7.12–7.00 (m, 2H), 6.88 (d, *J* = 8.1 Hz, 1H), 3.72 (s, 2H), 2.75–7.66 (m, 4H), 2.56 (t, *J* = 11.4 Hz, 2H), 2.34–2.39 (m, 6H), 2.18 (d, *J* = 5.4 Hz, 3H), 2.09 (d, *J* = 13.6 Hz, 2H), 1.81 (td, *J* = 12.5, 3.6 Hz, 2H). LCMS (*m*/*z*) 229.0 (M + H)^+^ *t*_*R*_ = 1.75 min.

#### 7-(3-Isopropylphenyl)-8-methyl-1′-(pyridin-2-ylmethyl)­spiro­[chromane-2,4′-piperidin]-4-one (**34**)

Prepared as described for **32**, brown oil. ^1^H NMR (CDCl_3_): δ 8.59 (d, *J* = 4.8 Hz, 1H), 7.87–7.71 (m, 3H), 7.40–7.31 (m, 2H), 7.27 (s, 1H), 7.16–7.06 (m, 2H), 6.95 (d, *J* = 8.1 Hz, 1H), 4.27 (br s, 2H), 3.49 (s, 2H), 3.32 (br s, 2H), 2.97 (h, *J* = 6.9 Hz, 1H), 2.81 (s, 2H), 2.54 (br s, 2H), 2.25 (d, *J* = 14.5 Hz, 2H), 2.08 (s, 3H), 1.29 (d, 6.9 Hz, 6H). LCMS (*m*/*z*) 441.3 (M + H)^+^ *t*_*R*_ = 1.90 min.

#### 8-Methyl-1′-(pyridin-2-ylmethyl)-7-(pyridin-3-yl)­spiro­[chromane-2,4′-piperidin]-4-one (**35**)

Prepared as described for **32**, orange powder. ^1^H NMR (CDCl_3_): δ 8.59 (dd, *J* = 4.9, 1.7 Hz, 1H), 8.54 (d, *J* = 2.3 Hz, 1H), 8.51 (d, *J* = 4.3 Hz, 1H), 7.73 (d, *J* = 8.1 Hz, 1H), 7.63–7.56 (m, 2H), 7.38–7.30 (m, 2H), 7.12 (dd, *J* = 7.5, 4.9 Hz, 1H), 6.83 (d, *J* = 8.1 Hz, 1H), 3.67 (s, 2H), 2.73–2.61 (m, 4H), 2.51 (dt, *J* = 10.6, 6.6 Hz, 2H), 2.14 (s, 3H), 2.05 (d, *J* = 11.9 Hz, 2H), 1.78 (dd, *J* = 12.5, 4.5 Hz, 2H). LCMS (*m*/*z*) 400.1 (M + H)^+^ *t*_*R*_ = 1.09 min.

#### 8-Methyl-1′-(pyridin-2-ylmethyl)-7-(pyridin-4-yl)­spiro­[chromane-2,4′-piperidin]-4-one (**36**)

Prepared as described for **32**, orange powder. ^1^H NMR (CDCl_3_): δ 8.69–8.63 (m, 2H), 8.53 (d, *J* = 4.9 Hz, 1H), 7.74 (d, *J* = 8.0 Hz, 1H), 7.62 (t, *J* = 7.7 Hz, 1H), 7.36 (d, *J* = 7.8 Hz, 1H), 7.24–7.18 (m, 2H), 7.14 (t, *J* = 6.3 Hz, 1H), 6.82 (d, *J* = 8.1 Hz, 1H), 3.68 (s, 2H), 2.73–2.66 (m, 4H), 2.52 (t, *J* = 11.4 Hz, 2H), 2.15 (s, 3H), 2.04 (t, *J* = 14.2 Hz, 2H), 1.80 (td, *J* = 12.4, 4.3 Hz, 2H). LCMS (*m*/*z*) 400.1 (M + H)^+^ *t*_*R*_ = 1.08 min.

#### 8-Methyl-7-(2-methylpyridin-4-yl)-1′-(pyridin-2-ylmethyl)­spiro­[chromane-2,4′-piperidin]-4-one (**37**)

Prepared as described for **32**, orange powder. ^1^H NMR (CDCl_3_): δ 8.61–8.45 (m, 2H), 7.73 (d, *J* = 8.1 Hz, 1H), 7.63 (t, *J* = 7.7 Hz, 1H), 7.37 (d, *J* = 7.8 Hz, 1H), 7.14 (t, *J* = 6.2 Hz, 1H), 7.08 (s, 1H), 7.02 (s, 1H), 6.81 (d, *J* = 8.1 Hz, 1H), 3.69 (s, 2H), 2.77–2.65 (m, 4H), 2.60 (s, 3H), 2.53 (t, *J* = 11.5 Hz, 2H), 2.15 (s, 3H), 2.07 (d, *J* = 13.8 Hz, 2H), 1.81 (t, *J* = 13.6 Hz, 2H). LCMS (*m*/*z*) 414.1 (M + H)^+^ *t*_*R*_ = 1.11 min.

#### 7-(2,6-Dimethylpyridin-4-yl)-8-methyl-1′-(pyridin-2-ylmethyl)­spiro­[chromane-2,4′-piperidin]-4-one (**38**)

Prepared as described for **32**, orange powder. ^1^H NMR (CDCl_3_): δ 8.55 (d, *J* = 4.9 Hz, 1H), 7.73 (d, *J* = 8.1 Hz, 1H), 7.64 (dt, *J* = 7.5, 3.9 Hz, 1H), 7.38 (d, *J* = 7.8 Hz, 1H), 7.16 (t, *J* = 6.1 Hz,, 1H), 6.89 (s, 2H), 6.80 (d, *J* = 8.1 Hz, 1H), 3.47 (s, 2H), 2.74–2.66 (m, 4H), 2.60–2.46 (m, 8H), 2.15 (s, 3H), 2.07 (d, *J* = 13.7 Hz, 2H), 1.81 (td, *J* = 12.9, 4.4 Hz, 2H). LCMS (*m*/*z*) 428.1 (M + H)^+^ *t*_*R*_ = 1.14 min.

#### 7-(2-Methoxypyridin-4-yl)-8-methyl-1′-(pyridin-2-ylmethyl)­spiro­[chromane-2,4′-piperidin]-4-one (**39**)

Prepared as described for **32**, brown crystals. ^1^H NMR (CDCl_3_): δ 8.58 (d, *J* = 4.9 Hz, 1H), 8.23 (d, *J* = 5.2 Hz, 1H), 7.76 (d, *J* = 8.2 Hz, 1H), 7.74–7.64 (m, 2H), 7.55–7.40 (m, 1H), 6.87 (d, *J* = 8.0 Hz, 1H), 6.80 (d, *J* = 5.2 Hz, 1H), 6.66 (s, 1H), 4.00 (s, 3H), 3.28 (br s, 2H), 2.77 (s, 2H), 2.51 (br s, 2H), 2.21–2.05 (m, 5H), 1.64 (br s, 4H). LCMS (*m*/*z*) 430.2 (M + H)^+^ *t*_*R*_ = 1.46 min.

#### 8-Methyl-7-(1-methyl-1*H*-pyrazol-4-yl)-1′-(pyridin-2-ylmethyl)­spiro­[chromane-2,4′-piperidin]-4-one (**40**)

Prepared as described for **32**, yellow solid. ^1^H NMR (CDCl_3_): δ 8.57 (d, *J* = 4.9 Hz, 1H), 7.70 (d, *J* = 8.2 Hz, 1H), 7.66 (d, *J* = 1.7 Hz, 1H), 7.64 (d, *J* = 0.8 Hz, 1H), 7.51 (s, 1H), 7.43 (d, *J* = 7.8 Hz, 1H), 7.18 (t, *J* = 6.3 Hz, 1H), 6.96 (d, *J* = 8.1 Hz, 1H), 3.98 (s, 3H), 3.76 (s, 2H), 2.74 (br s, 2H), 2.70 (s, 2H), 2.60 (br s, 2H), 2.32 (s, 3H), 2.09 (d, *J* = 13.6 Hz, 2H), 1.86 (br s, 2H). LCMS (*m*/*z*) 403.2 (M + H)^+^ *t*_*R*_ = 1.34 min.

#### 7-(1*H*-indol-5-yl)-8-methyl-1′-(pyridin-2-ylmethyl)­spiro­[chromane-2,4′-piperidin]-4-one (**41**)

Prepared as described for **32**, brown powder. ^1^H NMR (CDCl_3_): δ 8.58 (d, *J* = 4.9 Hz, 1H), 8.29 (s, 1H), 7.79–7.73 (m, 2H), 7.56 (s, 1H), 7.46 (d, *J* = 8.3 Hz, 1H), 7.32–7.28 (m, 2H), 7.13 (d, *J* = 8.3 Hz, 1H), 7.01 (d, *J* = 8.4 Hz, 1H), 6.61 (s, 1H), 4.28 (br s, 2H), 3.36 (br s, 2H), 2.78 (s, 2H), 2.33–2.04 (m, 5H), 1.78–1.45 (m, 4H). LCMS (*m*/*z*) 438.3 (M + H)^+^ *t*_*R*_ = 1.59 min.

#### 8-Methyl-1′-(pyridin-2-ylmethyl)-7-(quinolin-6-yl)­spiro­[chromane-2,4′-piperidin]-4-one (**42**)

Prepared as described for **32**, yellow powder. ^1^H NMR (CDCl_3_): δ 8.93 (dd, *J* = 4.3, 1.7 Hz, 1H), 8.54 (dd, *J* = 4.9, 1.9 Hz, 1H), 8.15 (d, *J* = 8.5 Hz, 1H), 7.82–7.72 (m, 3H), 7.63 (td, *J* = 7.7, 1.8 Hz, 1H), 7.41–7.35 (m, 2H), 7.32 (dd, *J* = 8.5, 4.2 Hz, 1H), 7.14 (dd, *J* = 7.5, 4.9 Hz, 1H), 6.88 (d, *J* = 8.0 Hz, 1H), 3.69 (s, 2H), 2.85–2.65 (m, 4H), 2.63–2.45 (m, 2H), 2.18–2.05 (m, 2H), 1.92 (s, 3H), 1.90–1.74 (m, 2H). LCMS (*m*/*z*) 450.1 (M + H)^+^ *t*_*R*_ = 1.21 min.

#### 8-Methyl-7-((6-methylpyridin-2-yl)­amino)-1′-(pyridin-2-ylmethyl)­spiro­[chromane-2,4′-piperidin]-4-one (**43**)

Prepared as described for **32**, yellow powder. ^1^H NMR (CDCl_3_): δ 8.59 (d, *J* = 4.5 Hz, 1H), 7.76–7.67 (m, 2H), 7.54–7.46 (m, 2H), 7.28–7.20 (m, 2H), 6.82 (d, *J* = 8.2 Hz, 1H), 6.74 (d, *J* = 7.4 Hz, 1H), 3.89 (br s, 2H), 2.85 (br s, 2H), 2.70 (s, 2H), 2.49 (s, 3H), 2.17 (s, 3H), 2.12 (br s, 2H), 2.01 (br s, 2H), 1.73 (br s, 2H). LCMS (*m*/*z*) 429.3.0 (M + H)^+^ *t*_*R*_ = 1.20 min.

#### *Tert*-Butyl 8-Methyl-7-(2-methylpyridin-4-yl)-4-oxospiro­[chromane-2,4′-piperidine]-1′-carboxylate (**I**-**12**)

Prepared as described for **26**, using intermediate **I**-**8**, brown powder, 1.707 g, 84%. ^1^H NMR (CDCl_3_): δ 8.57 (d, *J* = 5.2 Hz, 1H), 7.78 (d, *J* = 8.0 Hz, 1H), 7.13 (s, 1H), 7.08 (dd, *J* = 5.2, 1.6 Hz, 1H), 6.86 (d, *J* = 8.1 Hz, 1H), 3.95 (s, 2H), 3.17 (t, *J* = 13.3 Hz, 2H), 2.75 (s, 2H), 2.65 (s, 3H), 2.17 (s, 3H), 2.07 (d, *J* = 13.8 Hz, 2H), 1.65 (td, *J* = 12.5, 4.8 Hz, 2H) 1.46 (s, 9H). LCMS (*m*/*z*) 423.3 (M + H)^+^ *t*_*R*_ = 1.59 min.

#### *Tert*-Butyl 4-Hydroxy-8-methyl-7-(2-methylpyridin-4-yl)­spiro­[chromane-2,4′-piperidine]-1′-carboxylate (**I**-**13**)

Intermediate **I**-**13** (19.98 g, 1.0 eq, 47.3 mmol) was dissolved in MeOH (100 mL) and cooled to 0 °C, sodium borohydride (4.46 g, 2.5eq, 118.2 mmol) was added portion wise and the reaction was stirred for 10 min before warming to room temp and left to react for a further 4 h. At completion the reaction was diluted with water (150 mL) and extracted with DCM (2 × 50 mL) and EtOAc (50 mL). The organic layers were pooled, dried over anhydrous magnesium sulfate and concentrated in vacuo to give product as a pale yellow oil, 17.0 g, 84% which was carried on without further purification.

#### 8-Methyl-7-(2-methylpyridin-4-yl)­spiro­[chromane-2,4′-piperidine] (**I**-**14**)

Intermediate **I**-**13** (19.2 g, 1.0 eq, 45.2 mmol) was cooled to 0 °C and dissolved in TFA (50 mL) after 5 min triethylsilane (7.89 g, 1.5 eq, 67.9 mmol) was added dropwise and the reaction was heated to 80 °C for 6 h. At completion the reaction was diluted with water (100 mL), basified with NaOH (aq, 2M) to a pH of 10 and extracted with DCM (3 × 50 mL). The organic layers were pooled, dried over anhydrous magnesium sulfate and concentrated in vacuo to give crude product. Further purification was conducted by flash column chromatography running from 0 to 30% MeOH in DCM supplemented with 1% Et_3_N to give product as an off white solid 13.2 g, 94%. LCMS (*m*/*z*) 309.3 (M + H)^+^ *t*_*R*_ = 1.12 min.

#### 1′-((6-Chloropyridin-2-yl)­methyl)-8-methyl-7-(2-methylpyridin-4-yl)­spiro­[chromane-2,4′-piperidine] (**I**-**15**)

Prepared as described for **26**, using intermediate **I**-**14**, yellow solid. LCMS (*m*/*z*) 434.2 (M + H)^+^ *t*_*R*_ = 1.25 min.

#### 1′-((5-Bromopyridin-2-yl)­methyl)-8-methyl-7-(2-methylpyridin-4-yl)­spiro­[chromane-2,4′-piperidine] (**I**-**16**)

Prepared as described for **26**, using intermediate **I**-**14**, light yellow crystals, 1.3 g, 85%. LCMS (*m*/*z*) 478.2 (M + H)^+^ *t*_*R*_ = 1.24 min.

#### 8-Methyl-7-(2-methylpyridin-4-yl)-1′-(pyridin-2-ylmethyl)­spiro­[chromane-2,4′-piperidine] (**44**)

Prepared as described for **26**, using intermediate **I**-**14**. ^1^H NMR (CDCl_3_): δ 8.57 (d, *J* = 4.2 Hz, 1H), 8.50 (d, *J* = 5.1 Hz, 1H), 7.65 (td, *J* = 7.6, 1.8 Hz, 1H), 7.42 (d, *J* = 7.8 Hz, 1H), 7.17 (ddd, *J* = 7.5, 4.9, 1.2 Hz, 1H), 7.10 (s, 1H), 7.04 (dd, *J* = 5.2, 1.6 Hz, 1H), 6.95 (d, *J* = 7.8 Hz, 1H), 6.69 (d, *J* = 7.7 Hz, 1H), 3.73 (s, 2H), 2.81 (t, *J* = 6.8 Hz, 2H), 2.74 (br d, *J* = 11.1 Hz, 2H), 2.60 (s, 3H), 2.12 (s, 3H), 1.90–1.70 (m, 8H). LCMS (*m*/*z*) 400.30 (M + H)^+^ *t*_*R*_ = 1.17 min.

#### 1′-([2,3′-Bipyridin]-6-ylmethyl)-8-methyl-7-(2-methylpyridin-4-yl)­spiro­[chromane-2,4′-piperidine] (**45**)

Intermediate **I**-**15** (40.1 mg, 1.0 eq, 0.092 mmol), pyridine-3-boronic acid (17.5 mg, 1.5 eq, 0.138 mmol) and cesium carbonate (75.1 mg, 2.5 eq, 0.230 mmol) were dissolved in an 8:2 mix of dioxane/water (1 mL) and the mixture was purged with argon. Pd­(dppf)­Cl_2_·DCM (3.8 mg, 0.05 eq, 0.004 mmol) was added and the reaction was heated to 90 °C for 4 h. At completion the reaction was diluted with water (20 mL) and extracted with DCM (3 × 10 mL). The organic layers were combined, dried over anhydrous magnesium sulfate and concentrated in vacuo to give crude product. Further purification was conducted via reverse phase preparative HPLC with a gradient of 5–60% MeCN in water to give product as a yellow solid. ^1^H NMR (CDCl_3_): δ 8.50 (d, *J* = 5.1 Hz, 1H), 7.44 (dd, *J* = 8.4, 7.3 Hz, 1H), 7.10 (s, 1H), 7.05 (dd, *J* = 5.1, 1.6 Hz, 1H), 6.95 (d, *J* = 7.8 Hz, 1H), 6.77 (d, *J* = 7.3 Hz, 1H), 6.69 (d, *J* = 7.8 Hz, 1H), 6.50 (d, *J* = 8.4 Hz, 1H), 3.60 (s, 2H), 3.54 (t, *J* = 5.1 Hz, 4H), 2.80 (q, *J* = 6.8 Hz, 4H), 2.64–2.57 (m, 5H), 2.51 (t, *J* = 5.1 Hz, 4H), 2.33 (s, 3H), 2.10 (s, 3H), 1.91–1.66 (m, 6H). LCMS (*m*/*z*) 477.3 (M + H)^+^ *t*_*R*_ = 1.14 min.

#### 8-Methyl-1′-((6-(4-methylpiperazin-1-yl)­pyridin-2-yl)­methyl)-7-(2-methylpyridin-4-yl)­spiro­[chromane-2,4′-piperidine] (**46**)

Intermediate **I**-**15** (60.5 mg, 1.0 eq, 0.139 mmol), 1-methylpiperazine (18.2 mg, 1.3 eq, 0.182 mmol), cesium carbonate (136.2 mg, 3.0 eq, 0.418 mmol) and RuPhos (13.0 mg, 0.2 eq, 0.028 mmol) were dissolved in dioxane (1 mL) and the reaction was purged with argon. Pd_2_(dba)_3_ (6.3 mg, 0.05 eq, 0.07 mmol) was added and the reaction was heated to 100 °C for 16 h. At completion the reaction was diluted with water (20 mL) and extracted with DCM (3 × 10 mL). The organic layers were combined, dried over anhydrous magnesium sulfate and concentrated in vacuo to give crude product. Further purification was conducted via reverse phase preparative HPLC with a gradient of 5–60% MeCN in water to give product as a yellow solid 21.2 mg, 31%. ^1^H NMR (CDCl_3_): δ 8.50 (d, *J* = 5.1 Hz, 1H), 7.44 (dd, *J* = 8.4, 7.3 Hz, 1H), 7.10 (s, 1H), 7.05 (dd, *J* = 5.1, 1.6 Hz, 1H), 6.95 (d, *J* = 7.8 Hz, 1H), 6.77 (d, *J* = 7.3 Hz, 1H), 6.69 (d, *J* = 7.8 Hz, 1H), 6.50 (d, *J* = 8.4 Hz, 1H), 3.60 (s, 2H), 3.54 (t, *J* = 5.1 Hz, 4H), 2.85–2.74 (m, 4H), 2.59 (s, 3H), 2.51 (t, *J* = 5.1 Hz, 4H), 2.33 (s, 3H), 2.10 (s, 3H), 1.91–1.66 (m, 6H). LCMS (*m*/*z*) 498.3 (M + H)^+^ *t*_*R*_ = 1.17 min.

#### 1′-((6-(1*H*-pyrazol-4-yl)­pyridin-2-yl)­methyl)-8-methyl-7-(2-methylpyridin-4-yl)­spiro­[chromane-2,4′-piperidine] (**47**)

Prepared as described for **45**, white solid, 10.7 mg, 5%. ^1^H NMR (CDCl_3_): δ 8.50 (d, *J* = 5.0 Hz, 1H), 8.11 (s, 2H), 7.63 (t, *J* = 7.6 Hz, 1H), 7.38 (d, *J* = 7.8 Hz, 1H), 7.24 (s, 1H), 7.10 (s, 1H), 7.04 (d, *J* = 5.2 Hz, 1H), 6.96 (d, *J* = 7.8 Hz, 1H), 6.69 (d, *J* = 7.8 Hz, 1H), 3.79 (s, 2H), 2.91–2.75 (m, 4H), 2.72–2.62 (m, 2H), 2.60 (s, 3H), 2.11 (s, 3H), 1.94–1.75 (m, 6H). LCMS (*m*/*z*) 466.2 (M + H)^+^ *t*_*R*_ = 1.27 min.

#### *N,N*-dimethyl-1-(6-((8-methyl-7-(2-methylpyridin-4-yl)­spiro­[chromane-2,4′-piperidin]-1′-yl)­methyl)­pyridin-2-yl)­piperidin-4-amine (**48**)

Prepared as described for **46**, yellow oil, 21.8 mg, 37%. ^1^H NMR (CDCl_3_): δ 8.51 (d, *J* = 5.1 Hz, 1H), 7.43 (dd, *J* = 8.5, 7.3 Hz, 1H), 7.10 (s, 1H), 7.04 (dd, *J* = 5.1, 1.6 Hz, 1H), 6.96 (d, *J* = 7.8 Hz, 1H), 6.75 (d, *J* = 7.3 Hz, 1H), 6.69 (d, *J* = 7.8 Hz, 1H), 6.53 (d, *J* = 8.4 Hz, 1H), 4.37 (d, *J* = 12.8 Hz, 2H), 3.63 (s, 2H), 2.89–2.72 (m, 6H), 2.68–2.61 (m, 2H), 2.60 (s, 3H), 2.50–2.41 (m, 1H), 2.35 (s, 6H), 2.10 (s, 3H), 2.00–1.73 (m, 8H), 1.53 (qd, *J* = 12.1, 4.2 Hz, 2H). LCMS (*m*/*z*) 526.3 (M + H)^+^ *t*_*R*_ = 1.15 min.

#### (S)-1′-((6-(2,4-dimethylpiperazin-1-yl)­pyridin-2-yl)­methyl)-8-methyl-7-(2-methylpyridin-4-yl)­spiro­[chromane-2,4′-piperidine] (**49**)

Prepared as described for **46**, clear oil. ^1^H NMR (CDCl_3_): δ 8.50 (d, *J* = 5.1 Hz, 1H), 7.42 (dd, *J* = 8.5, 7.3 Hz, 1H), 7.10 (s, 1H), 7.04 (dd, *J* = 5.2, 1.6 Hz, 1H), 6.95 (d, *J* = 7.8 Hz, 1H), 6.74–6.66 (m, 2H), 6.44 (d, *J* = 8.5 Hz, 1H), 4.47 (s, 1H), 4.02 (d, *J* = 12.7 Hz, 1H), 3.60 (s, 2H), 3.11 (td, *J* = 12.4, 3.4 Hz, 1H), 2.92–2.67 (m, 7H), 2.62–2.57 (m, 6H), 2.28 (s, 3H), 2.27–2.20 (m, 1H), 2.10 (s, 3H), 2.09–2.01 (m, 1H), 1.91–1.66 (m, 7H). LCMS (*m*/*z*) 512.4.0 (M + H)^+^ *t*_*R*_ = 1.16 min.

#### 1-Methyl-4-(6-((8-methyl-7-(2-methylpyridin-4-yl)­spiro­[chromane-2,4′-piperidin]-1′-yl)­methyl)­pyridin-2-yl)­piperazin-2-one (**50**)

Prepared as described for **46**, yellow oil. ^1^H NMR (CDCl_3_): δ 8.50 (d, *J* = 5.1 Hz, 1H), 7.50 (dd, *J* = 8.4, 7.3 Hz, 1H), 7.10 (d, *J* = 1.6 Hz, 1H), 7.04 (dd, *J* = 5.1, 1.7 Hz, 1H), 6.96 (d, *J* = 7.8 Hz, 1H), 6.85 (d, *J* = 7.3 Hz, 1H), 6.69 (d, *J* = 7.8 Hz, 1H), 6.46 (d, *J* = 8.4 Hz, 1H), 4.09 (s, 2H), 3.90 (t, *J* = 5.4 Hz, 2H), 3.64 (s, 2H), 3.44 (t, *J* = 5.4 Hz, 2H), 3.03 (s, 3H), 2.87–2.77 (m, 4H), 2.66–2.60 (m, 2H), 2.59 (s, 3H), 2.10 (s, 3H), 1.93–1.72 (m, 6H). LCMS (*m*/*z*) 512.4.0 (M + H)^+^ *t*_*R*_ = 1.20 min.

#### 8-Methyl-7-(2-methylpyridin-4-yl)-1′-((6-morpholinopyridin-2-yl)­methyl)­spiro [Chromane-2,4′-piperidine] (**51**)

Prepared as described for **46**, orange solid. ^1^H NMR (CDCl_3_): δ 8.50 (d, *J* = 5.1 Hz, 1H), 7.47 (dd, *J* = 8.4, 7.3 Hz, 1H), 7.09 (d, *J* = 1.6 Hz, 1H), 7.03 (dd, *J* = 5.1, 1.7 Hz, 1H), 6.95 (d, *J* = 7.8 Hz, 1H), 6.82 (d, *J* = 7.3 Hz, 1H), 6.69 (d, *J* = 7.8 Hz, 1H), 6.50 (d, *J* = 8.4 Hz, 1H), 3.80 (t, *J* = 4.9 Hz, 4H), 3.68 (s, 2H), 3.47 (t, *J* = 4.9 Hz, 4H), 2.93 (t, *J* = 4.8 Hz, 2H), 2.90–2.84 (m, 2H), 2.81 (t, *J* = 6.8 Hz, 2H), 2.59 (s, 3H), 2.06 (s, 3H), 1.91–1.77 (m, 6H). LCMS (*m*/*z*) 485.3 (M + H)^+^ *t*_*R*_ = 1.26 min.

#### 8-Methyl-1′-((6-(4-methylpiperidin-1-yl)­pyridin-2-yl)­methyl)-7-(2-methylpyridin-4-yl)­spiro­[chromane-2,4′-piperidine] (**52**)

Prepared as described for **46**, yellow oil, 21.9 mg, 41%. ^1^H NMR (CDCl_3_): δ 8.50 (d, *J* = 5.1 Hz, 1H), 7.41 (dd, *J* = 8.5, 7.3 Hz, 1H), 7.10 (d, *J* = 1.6 Hz, 1H), 7.04 (dd, *J* = 5.1, 1.7 Hz, 1H), 6.96 (d, *J* = 7.8 Hz, 1H), 6.74–6.67 (m, 2H), 6.51 (d, *J* = 8.5 Hz, 1H), 4.26 (dt, *J* = 12.8, 3.3 Hz, 2H), 3.64 (s, 2H), 2.90–2.70 (m, 4H), 2.64 (t, *J* = 11.4 Hz, 1H), 2.60 (s, 3H), 2.09 (s, 3H), 1.90–1.75 (m, 6H), 1.70 (dd, *J* = 12.1, 2.2 Hz, 2H), 1.62–1.51 (m, 2H), 1.29–1.13 (m, 4H), 0.95 (d, *J* = 6.5 Hz, 3H). LCMS (*m*/*z*) 497.4 (M + H)^+^ *t*_*R*_ = 1.43 min.

#### 8-Methyl-1′-((5-(4-methylpiperazin-1-yl)­pyridin-2-yl)­methyl)-7-(2-methylpyridin-4-yl)­spiro­[chromane-2,4′-piperidine] (**53**)

Prepared as described for **46** using intermediate **I**-**16**, yellow oil. ^1^H NMR (CDCl_3_): δ 8.52 (d, *J* = 5.1 Hz, 1H), 8.28 (d, *J* = 2.9 Hz, 1H), 7.27 (d, *J* = 8.4 Hz, 1H), 7.18 (dd, *J* = 8.6, 2.9 Hz, 1H), 7.12 (s, 1H), 7.07 (dd, *J* = 5.1, 1.6 Hz, 1H), 6.97 (d, *J* = 7.8 Hz, 1H), 6.71 (d, *J* = 7.8 Hz, 1H), 3.65 (s, 2H), 3.24 (t, *J* = 5.0 Hz, 4H), 2.83 (t, *J* = 6.8 Hz, 2H), 2.74 (d, *J* = 11.2 Hz, 2H), 2.62 (s, 3H), 2.61–2.51 (m, 6H), 2.37 (s, 3H), 2.14 (s, 3H), 2.05 (s, 2H), 1.90–1.70 (m, 4H). LCMS (*m*/*z*) 498.3 (M + H)^+^ *t*_*R*_ = 1.12 min.

#### 1′-((5-(1*H*-pyrazol-4-yl)­pyridin-2-yl)­methyl)-8-methyl-7-(2-methylpyridin-4-yl)­spiro­[chromane-2,4′-piperidine] (**54**)

Prepared as described for **45** using intermediate **I**-**16**, white, solid, 5.2 mg, 2%. ^1^H NMR (CDCl_3_): δ 8.74 (d, *J* = 2.1 Hz, 1H), 8.51 (d, *J* = 5.0 Hz, 1H), 7.89 (s, 2H), 7.77 (dd, *J* = 8.0, 2.4 Hz, 1H), 7.47 (d, *J* = 8.0 Hz, 1H), 7.10 (s, 1H), 7.05 (d, *J* = 5.2 Hz, 1H), 6.96 (d, *J* = 7.8 Hz, 1H), 6.70 (d, *J* = 7.8 Hz, 1H), 3.79 (s, 2H), 2.82 (t, *J* = 6.8 Hz, 4H), 2.60 (s, 3H), 2.11 (s, 3H), 2.06–2.02 (m, 2H), 1.91–1.78 (m, 6H). LCMS (*m*/*z*) 466.2 (M + H)^+^ *t*_*R*_ = 1.25 min.

#### Methyl 6-((8-methyl-7-(2-methylpyridin-4-yl)­spiro­[chromane-2,4′-piperidin]-1′-yl)­meth-yl)­nicotinate (**I**-**17**)

Prepared as described for **26**, using intermediate **I**-**14** off white solid. ^1^H NMR (CDCl_3_): δ 9.16 (dd, *J* = 2.2, 0.8 Hz, 1H), 8.50 (d, *J* = 5.1 Hz, 1H), 8.26 (dd, *J* = 8.1, 2.2 Hz, 1H), 7.56 (d, *J* = 8.1 Hz, 1H), 7.10 (d, *J* = 1.6 Hz, 1H), 7.04 (dd, *J* = 5.1, 1.7 Hz, 1H), 6.96 (d, *J* = 7.8 Hz, 1H), 6.69 (d, *J* = 7.8 Hz, 1H), 3.94 (s, 3H), 3.80 (s, 2H), 2.81 (t, *J* = 6.8 Hz, 2H), 2.73 (d, *J* = 10.0 Hz, 2H), 2.63 (d, *J* = 11.4 Hz, 2H), 2.59 (s, 3H), 2.13 (s, 3H), 1.91–1.73 (m, 6H).

#### 6-((8-Methyl-7-(2-methylpyridin-4-yl)­spiro­[chromane-2,4′-piperidin]-1′-yl)­methyl)-*N*-(thiazol-4-ylmethyl)­nicotinamide (**55**)

Intermediate **I-17** (102 mg, 1.0 eq, 0.102 mmol) and lithium hydroxide (54.0 mg, 10.0 eq, 53.4 mmol) were dissolved in a 1:1:1 mixture of THF/water/methanol (2 mL) and the reaction was heated to 40 °C for 1 h. At completion the reaction diluted with water (20 mL) basified with NaOH (aq, 2 M) and washed with DCM, the aqueous layer was then acidified and extracted with DCM (2 × 10 mL) and EtOAc (10 mL). The organic layers were pooled, dried over anhydrous magnesium sulfate and concentrated in vacuo. The resulting solid was dissolved in DMF (2 mL), thiazol-4-ylmethanamine (38.6 mg, 1.5 eq, 0.338 mmol), HATU (171.0 mg, 0.450 mmol) and DIPEA (87.6 mg, 3.0 eq, 0.676 mmol) were added and the reaction was stirred at room temp for 4 h. At completion the reaction was diluted with water (20 mL) and extracted with DCM (2 × 10 mL) and EtOAc (10 mL). The organic layers were pooled, dried over anhydrous magnesium sulfate and concentrated in vacuo to give crude product. Further purification was conducted via reverse phase preparative HPLC with a gradient of 5–60% MeCN in water to give product as an off white solid, 65.2 mg, 54%. ^1^H NMR (CDCl_3_): δ 9.00 (s, 1H), 8.79 (d, *J* = 2.0 Hz, 1H), 8.52 (d, *J* = 5.1 Hz, 1H), 8.15 (d, *J* = 8.0 Hz, 1H), 7.31 (d, *J* = 2.0 Hz, 1H), 7.09 (s, 1H), 7.08–6.93 (m, 2H), 6.73 (d, *J* = 7.8 Hz, 1H), 4.80 (d, *J* = 5.4 Hz, 2H), 4.10 (s, 2H), 3.10 (s, 2H), 2.83 (t, *J* = 6.9 Hz, 3H), 2.61 (s, 3H), 2.10 (s, 3H), 1.99–1.82 (m, 4H), 1.67 (s, 2H), 1.25 (s, 3H). LCMS (*m*/*z*) 540.3 (M + H)^+^ *t*_*R*_ = 1.24 min.

#### Methyl 2-Chloro-6-((8-methyl-7-(2-methylpyridin-4-yl)­spiro­[chromane-2,4′-piperidin]-1′-yl)­methyl)­nicotinate (**I**-**18**)

Prepared as described for **26**, using intermediate **I**-**14**, clear yellow oil, 0.50 mg, 75%. LCMS (*m*/*z*) 492.3 (M + H)^+^ *t*_*R*_ = 1.23 min.

#### Methyl 6-((8-methyl-7-(2-methylpyridin-4-yl)­spiro­[chromane-2,4′-piperidin]-1′-yl)­meth-yl)-2-(4-methylpiperazin-1-yl)­nicotinate (**56**)

Prepared as described for **46**, using intermediate **I**-**18**, white solid, 29.5 mg, 43%. ^1^H NMR (CDCl_3_): δ 8.51 (d, *J* = 5.1 Hz, 1H), 8.01 (d, *J* = 7.8 Hz, 1H), 7.10 (s, 1H), 7.04 (d, *J* = 4.9 Hz, 1H), 6.99–6.93 (m, 2H), 6.70 (d, *J* = 7.8 Hz, 1H), 3.86 (s, 3H), 3.69 (s, 2H), 3.51 (s, 4H), 2.82 (t, *J* = 6.7 Hz, 4H), 2.66 (s, 4H), 2.60 (s, 3H), 2.43 (s, 3H), 2.15 (d, *J* = 13.0 Hz, 2H), 2.09 (s, 3H), 1.85 (dd, *J* = 12.9, 5.9 Hz, 6H). LCMS (*m*/*z*) 556.3 (M + H)^+^ *t*_*R*_ = 1.13 min.

#### 6-((8-Methyl-7-(2-methylpyridin-4-yl)­spiro­[chromane-2,4′-piperidin]-1′-yl)­methyl)-2-(4-methylpiperazin-1-yl)-*N*-(thiazol-4-ylmethyl)­nicotinamide (**57**)

Prepared as described for **55**, using intermediate **56**, yellow solid. ^1^H NMR (CDCl_3_): δ 8.83 (d, *J* = 2.0 Hz, 1H), 8.51 (d, *J* = 5.1 Hz, 1H), 8.29 (d, *J* = 7.8 Hz, 1H), 7.33–7.28 (m, 2H), 7.09 (s, 1H), 7.03 (d, *J* = 5.0 Hz, 1H), 6.97 (d, *J* = 7.8 Hz, 1H), 6.71 (d, *J* = 7.7 Hz, 1H), 4.78 (d, *J* = 5.2 Hz, 2H), 3.80 (br s, 2H), 3.28 (br s, 4H), 2.82 (t, *J* = 6.8 Hz, 4H), 2.62–2.57 (m, 6H), 2.39 (s, 2H), 2.11–2.04 (m, 4H), 1.95–1.82 (m, 6H), 1.76–1.54 (m, 4H). LCMS (*m*/*z*) 638.3 (M + H)^+^ *t*_*R*_ = 1.19 min.

### Protein Expression and Purification

The gene containing the ubiquitin-like domain and the catalytic core of SARS-CoV-2 PL^Pro^ (residues 1–315) was synthesized with codon optimization for *Escherichia coli* and cloned in the pET28a­(+) vector by GenScript. We made PL^Pro^ constructs to optimize the protein for NMR-based fragment screen (residues 71–314 with C111S and C270S), X-ray crystallography (residues 1–314 with C111S and C270S) and the enzymatic assays (residues 1–315 with C270S) were obtained by site mutagenesis. All PL^Pro^ plasmids were transformed into the BL21­(DE3) strain *E. coli*. The bacteria were cultured in Luria–Bertani broth or M9 minimal media containing ^15^NH_4_Cl supplemented with 50 mg/mL Kanamycin at 37 °C until the optimal density at 600 nm reached 0.8 before inducing protein expression by the addition of 0.1 mM IPTG and 0.1 mM ZnCl_2_ at 18 °C for 20 h. The cell pellet was harvested by centrifugation at 5000 g for 15 min, resuspended in lysis buffer (50 mM Tris pH 7.0, 500 mM NaCl, 5% glycerol, 10 mM imidazole, 5 mM BME, 0.1% Triton X-100 and 1 mM PMSF), and lysed in APV2000 lab homogenizer (SPX flow). Cell lysate was centrifuged at 15,000 g for 45 min and loaded onto HisTrap FF column (Cytiva). The column was washed with 10 column volumes of Buffer A (50 mM Tris pH 7.0, 500 mM NaCl, 5% glycerol, 10 mM imidazole, 5 mM BME) and eluted with Buffer B (50 mM Tris pH 7.0, 500 mM NaCl, 5% glycerol, 500 mM imidazole, 5 mM BME) using a linear gradient program from 0 to 100% Buffer B over 10 column volumes. To the fractions containing PL^Pro^, Thrombin was added to remove the 6xHis tag and dialyzed against Buffer A without imidazole overnight. Then, Tag-cleaved PL^Pro^ was loaded on a HisTrap column. The flowthrough was concentrated and subjected to HiLoad 26/600 Superdex75 pg (Cytiva) and eluted using Buffer C (20 mM HEPES pH 7.0, 150 mM NaCl, 3 mM DTT) for the NMR-based fragment screen or Buffer D (25 mM Tris pH 7.0, 150 mM NaCl, 3 mM DTT) for X-ray crystallography and the enzymatic assays. Protein concentration was quantified by the Pierce 660 nm assay (ThermoFisher).

### NMR Experiments

All NMR experiments were performed at 25 °C using a 600 MHz Bruker Avance III spectrometer equipped with a 5 mm single-axis x-gradient cryoprobe and a Bruker SampleJet. Gradient-enhanced, two-dimensional ^1^H–^15^N heteronuclear multiple-quantum coherence (SOFAST-HMQC) spectra of PL^Pro^ were recorded using 24 scans of 12 min acquisition times and analyzed using Topspin 4.1.4 (Bruker). Our in-house fragment library of 13,824 compounds was screened as mixtures of 12 fragments prepared in 12 96-well plates. Each NMR sample was made of 15 mM of ^15^N-labeled PL^Pro^, 800 μM of each fragment, and 5% DMSO-*d*_6_ for spectrometer locking in 5 mm-diameter NMR tubes. Hit mixtures were identified by comparing the chemical shifts of the backbone resonances to a ligand-free PL^Pro^ spectrum and then deconvoluted by screening individual fragments.

SOFAST-HMQC titration experiments were used to determine binding affinity of the fragment hits identified from the screen. The changes in ^1^H–^15^N chemical shifts of backbone resonances upon the addition of increasing concentrations of the fragments (0.0625–2 mM) were analyzed. The binding affinities (K_d_s) of the fragments were calculated using the Hill’s equation model in Prism 10 (GraphPad).

### Crystallization and Data Collection

Compounds **11**, **17** and **27**. Seven mg/mL PL^Pro^ was mixed with a 200 mM DMSO stock of the desired fragments to a final concentration of 5 mM fragment and 2.5% DMSO and incubated at 4 °C overnight. Hanging drops were set up in a 1:1 ratio of protein + ligand/crystallization solution (0.2 mM sodium citrate and 15–25% PEG-3350) and incubated at 18 °C allowing vapor diffusion against the corresponding reservoir solution. The crystals were cryo-protected in mother liquor supplemented with 20% ethylene glycol before being flash-frozen in liquid nitrogen. Compounds **37**, **46**, **47** and **53**. Twelve mg/mL PL^Pro^ in buffer (25 mM TRIS pH = 7.0, 50 mM NaCl and 10 mM DTT) was incubated with various ligands (100 mM DMSO stocks, final concentration of 0.5–1 mM) at room temperature for 1 h then centrifuged to remove insoluble precipitate. Hanging drops were set up in a 1:1 ratio of protein + ligand/crystallization solution (50 mM HEPES pH = 7.0, Tryptone 2–4% w/v and 10–16% PEG-3350) and incubated at 18 °C allowing vapor diffusion against the corresponding reservoir solution. Large diamond shaped crystal appeared overnight or within 2 days of setup belonging to space group P 6_5_22. Ligands that failed to cocrystallize within 3 days of tray setup underwent competitive soaking with seed ligand **37**. Co-crystals of ligand **37** were transferred into a soaking drop of crystallization solution (50 mM HEPES pH = 7.0, Tryptone 2% w/v and 13% PEG-3350) supplemented with 10 mM ligand and incubated at 18 °C for 3 days. Following soaking, crystals were transferred to ligand free cryo and incubated for 5 min to wash out DMSO and improve crystal quality. All crystals were cryoprotected in mother liquor supplemented with 20% ethylene glycol or 20% glycerol before being flash cooled in liquid nitrogen. Data sets were acquired at 100 K on the Life Sciences Collaborative Access Team (LS-CAT) Sector-21 beamlines at the Advanced Photon Source (APS), Argonne National Laboratory or the Berkeley Center for Structural Biology (BSB) 8.2.2 beamline at the ALS using a Pilatus3 2 M detector. Diffraction data were indexed and integrated with XDS and scaled with aimless. Phasing was accomplished by molecular replacement with PhaserMR using the structure of SARS-CoV-2 PL^pro^ with C111S (PDB:6WRH) as starting model. Ligand models were built by elbow and manually added to the corresponding electron density. PL^Pro^-ligand cocrystal structures were determined by several cycles of refinement using Phenix and manual modeling with Coot.

### RLKGG Enzymatic Assay

All compounds were stored in 10 mM stock in DMSO. Dose responses of the compounds were generated using an ECHO 555 Liquid Handler (Labcyte, Inc.) in a 384 well black polystyrene flat bottom plate with a nonbinding surface (Corning p/n 3575). 10 μL of recombinant PL^Pro^ was added at a concentration of 200 nM and incubated for 30 min, followed by the addition of 10 μL of Ac-RLKGG-AMC at a concentration of 60 μM (The final concentrations of enzyme and substrate are 80 nM and 30 μM, respectively, with a DMSO concentration of 5%). AMC product formation was measured at 5 and 15 min, and the enzyme activity was expressed as moles AMC L^–1^sec^–1^. GRL-0617 served as a PL^Pro^ positive control inhibitor (Selleckchem). Positive control wells contained enzyme, substrate, and vehicle, while negative control wells had substrate and vehicle minus the enzyme. The dose range for the test compounds was 500 μM-0.98 μM or 100 μM-0.026 μM for low and high affinity compounds, respectively.

### A549 Cellular Antiviral Assay

3000 A549-ACE2 cells grown in RPMI supplemented with 1% penicillin/streptomycin, 2 mM l-glutamine, and 10% FBS were plated per well of a 384 well assay plate (Corning 3764). The next day, 50 nL of drug suspended in DMSO was added as an 8 pt dose response with 3-fold dilutions between test concentrations in triplicate, starting at 50–100 μM final concentration. The negative control (0.2% DMSO, n = 32) and positive control (10 μM Remdesivir, n = 32) were included on each assay plate. Cells were pretreated with controls and test drugs for 2 h prior to infection. In BSL3 containment, SARS-CoV-2 (isolate USA WA1/2020) diluted in serum free growth medium was added to plates to achieve an MOI = 0.5. Cells were incubated continuously with drugs and SARS-CoV2 for 24 h. Cells were fixed with 4% formaldehyde for 15 min at room temperature, washed 3X with PBS, blocked with 2% BSA (W/V) in PBS supplemented with 0.1% triton-x-100 (PBST) and incubated with primary antibody SPIKE (sotrovimab) diluted in PBST overnight at 4 °C. Cells were washed 3X with PBST and incubated with a secondary antibody (antimouse Alexa 488) and 10 μg/mL Hoechst 33342 for 1 h at room temperature. Cells were washed 3X with PBST and imaged on an automated microscope (ImageXpress Micro, Molecular Devices) at 10X, four sites per well. The total number of cells (Hoechst-stained nuclei count) and the number of infected (SPIKE+) cells were measured using the cell scoring module (MetaXpress 6.7.0), and the percentage of infected cells (SPIKE + cells/cell number) per well was calculated. SARS-CoV-2 infection at each drug concentration was normalized to aggregated DMSO plate control wells and expressed as percentage-of-control (POC = % Infection sample/Avg % Infection DMSO cont). A nonlinear regression curve fit analysis (CBIS) of POC Infection and cell viability versus the log10 transformed concentration values was used to determine the IC_50_/IC_90_ values for Infection and CC_50_ values for cell viability. Selectivity index (SI) was calculated as a ratio of drug’s CC_50_ and IC_50_ values (SI = CC_50_/IC_50_).

### Viral Passaging Experiments

A549 cells were plated at 2 × 10^5^ cells per well in 12-well plates 24 h before infection. Cells were then infected with wild type SARS-CoV-2 infectious clone based on the WA1 strain (SARS-CoV-2 WT) (GenBank MT461669.1) (PMCID: PMC7250779) at an MOI of 0.01 PFU/cell, and cells were incubated for 30 min at 37 °C prior to the addition of 1 μM **46** or 0.01% DMSO in cell medium, passage 0 (P0). Infected cells were treated with **46** in six independent parallel lineages and with DMSO in three independent parallel lineages. Infected cell supernatants were harvested when approximately 50–70% of the cell monolayers were engaged in cytopathic effect (CPE), P1. P1 virus stocks were then blindly passaged onto 2 × 10^5^ A549 cells using 20 μL of the 1 mL total sample. Cells were treated with increasing concentrations of **46** as determined by CPE acceleration. **46** passage lineages were terminated after the collection of P9 and 7 μM of compound. DMSO passage lineages were terminated after P7. Infected cell monolayers producing **46** P9 lineages or DMSO P7 lineages were harvested in TRIzol reagent, and viral RNA was extracted by chloroform extraction and purified using the KingFisher MagMAX Viral/Pathogen Nucleic Acid Isolation Kit (Thermo).

## Supplementary Material





## Notes

Example HMQC shift patterns and K_D_ titration curves for fragments **10** and **11**; X-ray refinement statistics of compounds, 11, 17, 27, 37, 46, 47, and 53; X-ray omit and electron density maps of compounds, 11, 17, 27, 37, 46, 47, and 53; Cellular antiviral activity, cytotoxicity and selectivity index for compounds **44**–**55**; Initial profiling of compound **46** (PDF)
Molecular formula strings (CSV)

## Abbreviations used:

CC_50_: half maximal cytotoxic concentration
DIPEA: diisopropylethylamine
EtOAc: ethyl acetate
Et_3_SiH: triethylsilane
HATU: hexafluorophosphate azabenzotriazole tetramethyl uronium
HEPES: 4-(2-hydroxyethyl)-1-piperazineethanesulfonic acid
MeCN: acetonitrile
MeOH: methanol
PEG: polyethylene glycol
PL^Pro^: Papain like protease
TEA: triethylamine
RT: retention time
SARS-CoV-2: Severe acute respiratory syndrome coronavirus 2
STAB: sodium triacetoxyborohydride
TRIS: *tris*(hydroxymethyl)­aminomethane
