# Supplementary material for: Discovery of Fragment-Based Inhibitors of SARS-CoV‑2 PLPro
Source: J Med Chem. 2026 Jan 12;69(2):1419–33. doi: 10.1021/acs.jmedchem.5c02832 (PMC12833870; doi:10.1021/acs.jmedchem.5c02832)
Supplement: Supplementary file 1 [file jm5c02832_si_001.pdf]

Supporting Information

Discovery of Fragment-Based Inhibitors of SARS-CoV-2 PL^{Pro}

Qiangqiang Wei†[1], Ashley J. Taylor†[1], Mahesh Angadrao Barmade[1], Kevin B. Teuscher[1], Somanath Chowdhury[1], Chideraa Apakama[1], Jordan Anderson-Daniels[2], Zhu Yongqing[3], David C. Schultz[3], Tyson A. Rietz[1], Taylor M. South[1], Mackenzie M. Crow[1], Bin Zhao[1], Kangsa Amporndanai[1], John L. Sensintaffar[1], Jason Phan[1], Sara Cherry[3], Mark Denison[2], Taekyu Lee[1], Stephen W. Fesik*[1,4,5].

†Co-first authors
*Corresponding author

[1] Department of Biochemistry, Vanderbilt University School of Medicine, Nashville, Tennessee 37232-0146, United States.
[2] Department of Pathology, Microbiology, and Immunology, Vanderbilt University Medical Center, Nashville, Tennessee 37232, United States
[3] Department of Pathology and Laboratory Medicine, University of Pennsylvania, Philadelphia, Pennsylvania 19104 United States
[4] Department of Pharmacology, Vanderbilt University School of Medicine, Nashville, Tennessee, 37232-6600, United States.
[5] Department of Chemistry, Vanderbilt University, Nashville, Tennessee, 37235-0146, United States.

Corresponding Author Contact

Stephen W. Fesik
Phone: +1 (615) 322-6303; Fax: +1 (615) 875-3236;
Email: Stephen.fesik@vanderbilt.edu

Table of Contents

Example NMR HMQC shift patterns for S3/4 subsites and zinc finger binders	S2
HMQC NMR titrations for hit fragments **10** and **11**	S2
X-ray data collection and refinement statistics	S3
Electron density and omit maps for submitted X-ray structures	S6
Cellular activity of compounds **44-55**	S8
Initial profiling of Compound **46**	S8

Example NMR HMQC shift patterns for S3/4 subsites and zinc finger binders

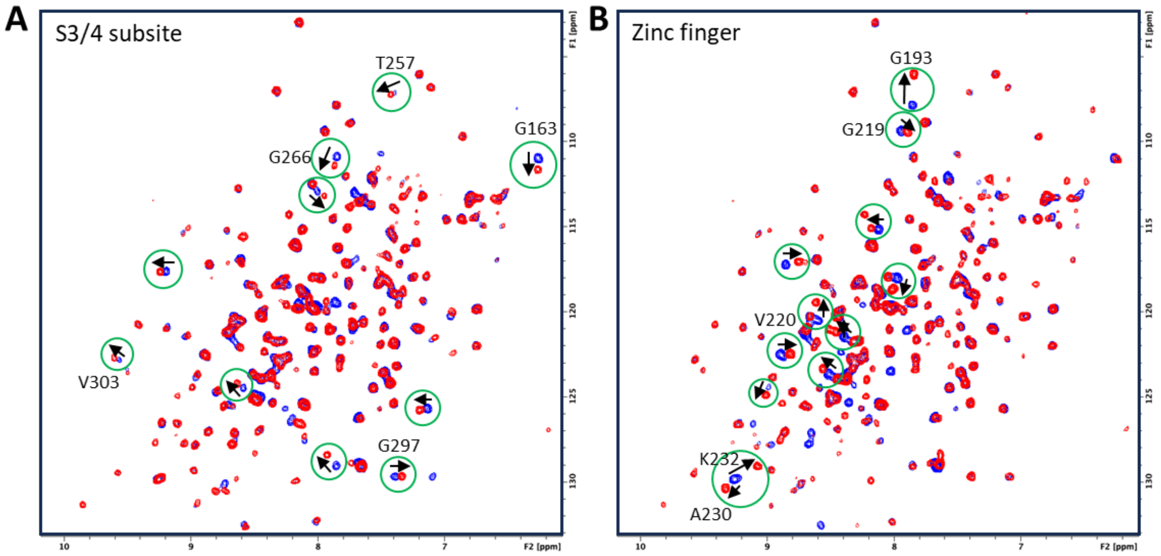

Figure S1. ^{1}H-^{15}N SOFAST HMQC spectra of PL^{Pro} without (blue) and with (red) 0.8 mM fragment, distinct perturbation patterns are observed for ligands which bind at the S3/4 subsite (A) and zinc finger region (B)

HMQC NMR titrations for hit fragments 10 and 11

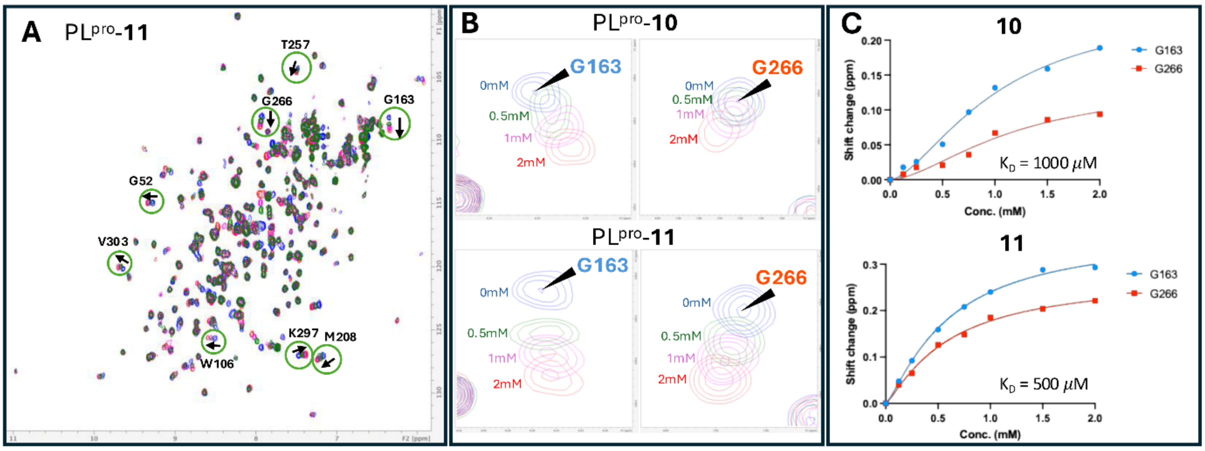

Figure S2. Chemical shift perturbations and affinity measurement for fragments **10** and **11**. (A) ^{1}H-^{15}N SOFAST-HMQC spectrum of PL^{Pro} without (blue) and with 0.5 mM (green), 1.0 mM (pink) and 2.0 mM (red) fragment **10** with key chemical shifts labelled. (B) Zoom in on select peak shifts used for K_D determination. (C) Titration curves of fragments **10** and **11**. Reported K_ds were average K_ds from several peak shifts.

X-ray data collection and refinement statistics

Table S1. X-ray data collection and refinement statistics for fragments bound to PL^{pro}.

	**Compound 11**<br>**9PUH**	**Compound 17**<br>**9PUJ**	**Compound 27**<br>**9PUY**
Resolution range	35.62 - 2.39<br>(2.475 - 2.39)	29.6 – 2.0<br>(2.03 - 2.0)	30.0-1.75<br>(1.78-1.75)
Space group	P 21 21 21	P 61 2 2	P 21 21 21
Unit cell<br>a, b, c (Å)<br>α, β, γ (°)	 99.553, 101.987, 128.375<br>90, 90, 90	 102.548, 102.548, 139.948<br>90, 90, 120	 98.269, 118.978, 124.824<br>90, 90, 90
Total reflections	1420701 (4499)	2704507 (3022)	6205214
Unique reflections	52105 (4874)	29686 (2853)	147824 (14404)
Multiplicity	12.6 (6.1)	24.0 (24.5)	6.3 (6.0)
Completeness (%)	99.35 (99.15)	99.03 (97.54)	99.43 (98.84)
Mean I/sigma(I)	27.1 (1.93)	29.3 (3.30)	20.6 (2.58)
Wilson B-factor	39.67	35.81	17.27
R-merge	0.043 (0.560)	0.095 (1.390)	0.129 (1.286)
R-meas	0.045 (0.610)	0.097 (1.419)	0.135 (1.345)
R-pim	0.012 (0.231)	0.020 (0.286)	0.038 (0.390)
CC1/2	0.99 (0.38)	0.999 (0.882)	0.998 (0.872)
Reflections used in refinement	52087 (4874)	29684 (2853)	146849 (14398)
Reflections used for R-free	1996 (188)	1444 (146)	7352 (734)
R-work	0.1925 (0.2730)	0.1971 (0.2504)	0.1705 (0.2141)
R-free	0.2446 (0.3230)	0.2344 (0.2852)	0.2050 (0.2549)
Number of non-hydrogen atoms	7532	2577	11722
macromolecules	7204	2369	9912
ligands	37	22	134
solvent	291	186	1676
Protein residues	941	307	1275
RMS(bonds)	0.007	0.007	0.006
RMS(angles)	0.87	0.84	0.86
Ramachandran favored (%)	95.19	97.38	97.16
Ramachandran allowed (%)	4.49	2.62	2.84
Ramachandran outliers (%)	0.32	0.00	0.00
Rotamer outliers (%)	0.94	0.81	0.00
Clashscore	4.36	3.24	3.70
Average B-factor	48.58	38.49	22.23
macromolecules	48.88	38.28	20.79
ligands	37.81	31.70	23.73
solvent	42.48	41.99	30.67

Statistics for the highest-resolution shell are shown in parentheses.

	Compound 37<br>9PV6	Compound 46<br>9PV9	Compound 47<br>9PVI
Resolution range	231.56 - 1.45 (1.47 - 1.45)	45.97 - 2.0 (2.05 - 2.0)	33.8 - 1.8 (1.84 - 1.8)
Space group	P 65 2 2	P 65 2 2	P 65 2 2
Unit cell<br>a, b, c (Å)<br>α, β, γ (º)	 78.405, 78.405, 231.561<br>90, 90, 120	 78.3, 78.3, 229.868<br>90, 90, 120	 79.3467, 79.3467, 232.837<br>90, 90, 120
Total reflections	2974789 (140765)	565194 (42234)	1616378 (95752)
Unique reflections	75599 (3649)	29258 (2114)	41339 (2392)
Multiplicity	39.3 (38.6)	19.3 (20.0)	39.1 (40.0)
Completeness (%)	100 (100)	100 (100)	100 (100)
Mean I/sigma(I)	23.0 (1.5)	34.7 (3.4)	20.2 (1.6)
Wilson B-factor	21.79	40.41	36.49
R-merge	0.086 (2.826)	0.052 (1.006)	0.094 (2.308)
R-meas	0.087 (2.864)	0.055 (1.057)	0.095 (2.337)
R-pim	0.014 (0.460)	0.013 (0.235)	0.015 (0.366)
CC1/2	1 (0.741)	1 (0.920)	0.999 (0.939)
Reflections used in refinement	75436 (7357)	29159 (2833)	41170 (4005)
Reflections used for R-free	3821 (389)	1471 (133)	2030 (179)
R-work	0.1607 (0.2262)	0.2102 (0.2676)	0.2238 (0.3041)
R-free	0.1972 (0.2754)	0.2371 (0.3304)	0.2561 (0.3593)
Number of non-hydrogen atoms	2814	2560	2563
macromolecules	2432	2398	2390
ligands	71	44	44
solvent	311	118	129
Protein residues	312	312	305
RMS(bonds)	0.007	0.007	0.007
RMS(angles)	1.11	1.06	0.93
Ramachandran favored (%)	96.45	95.16	96.68
Ramachandran allowed (%)	2.90	4.19	3.32
Ramachandran outliers (%)	0.65	0.65	0.00
Rotamer outliers (%)	0.79	2.41	1.99
Clashscore	3.35	3.85	2.56
Average B-factor	34.80	47.55	47.62
macromolecules	33.96	47.62	47.74
ligands	41.47	48.80	45.42
solvent	39.84	45.68	46.14

Statistics for the highest-resolution shell are shown in parentheses.

	**Compound 53**<br>**9PVK**
Resolution range	46.29 - 1.8 (1.84 - 1.8)
Space group	P 65 2 2
Unit cell<br>a, b, c (Å)<br>α, β, γ (°)	 78.167, 78.167, 231.464<br>90, 90, 120
Total reflections	806929 (48242)
Unique reflections	39922 (2311)
Multiplicity	20.2 (20.9)
Completeness (%)	100 (100)
Mean I/sigma(I)	41.5 (3.1)
Wilson B-factor	32.56
R-merge	0.045 (1.244)
R-meas	0.046 (1.274)
R-pim	0.010 (0.274)
CC1/2	1 (0.89)
Reflections used in refinement	39811 (3894)
Reflections used for R-free	1906 (191)
R-work	0.2095 (0.2959)
R-free	0.2470 (0.3489)
Number of non-hydrogen atoms	2675
macromolecules	2414
ligands	50
solvent	211
Protein residues	314
RMS(bonds)	0.009
RMS(angles)	1.42
Ramachandran favored (%)	95.19
Ramachandran allowed (%)	4.49
Ramachandran outliers (%)	0.32
Rotamer outliers (%)	0.40
Clashscore	5.06
Average B-factor	39.37
macromolecules	39.17
ligands	38.70
solvent	41.82

Statistics for the highest-resolution shell are shown in parentheses.

Electron density and omit maps of submitted X-ray structures

Table S2. Crystal structures of PLPro complexed with various ligands (orange sticks) with surface shown in grey and nearby residues as green lines. Composite omit maps: Fo-Fc positive (green mesh) and negative (red mesh) shown at 3.0 σ carved 2.0 Å around the ligand of interest. Electron density maps: 2Fo-Fc (blue mesh) and Fo-Fc positive (green mesh) and negative (red mesh) shown at 1.5, 3.0 σ respectively carved 2.0 Å around the ligand of interest.

	Omit map	Electron density map
Compound 11<br>9PUH	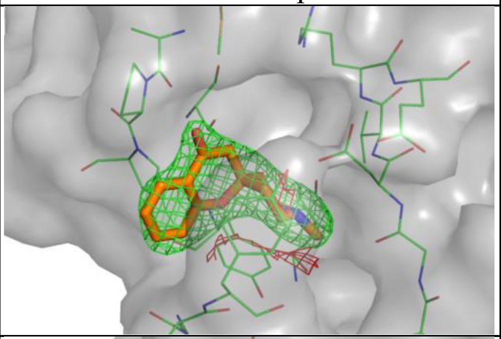	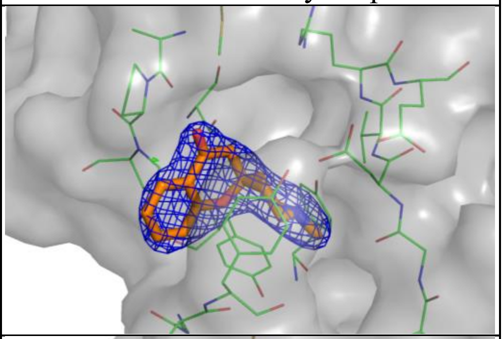
Compound 17<br>9PUJ	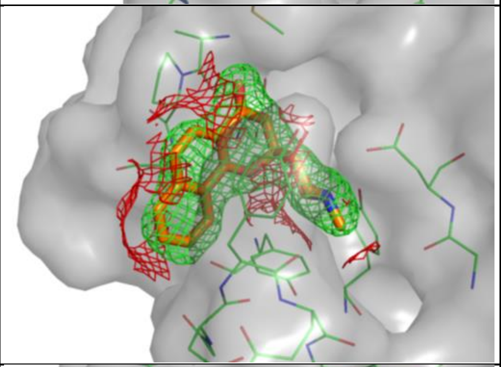	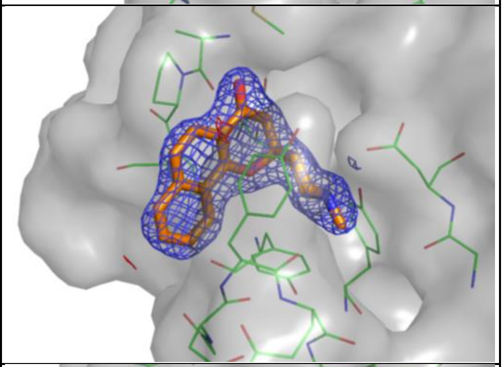
Compound 27<br>9PUY	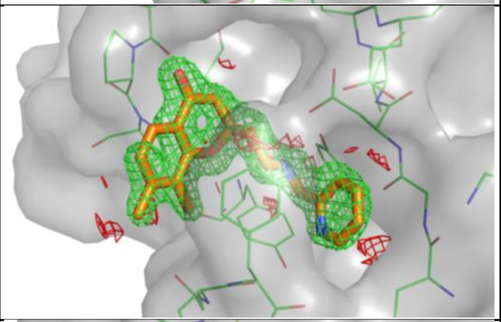	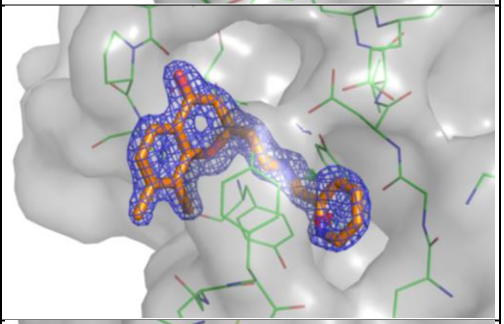
Compound 37<br>9PV6	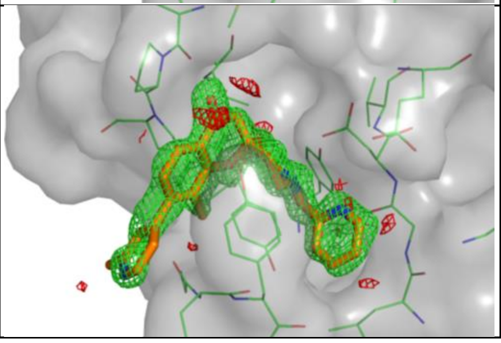	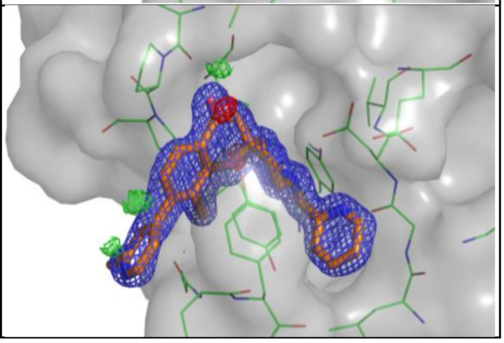

Compound 46<br>9PV9	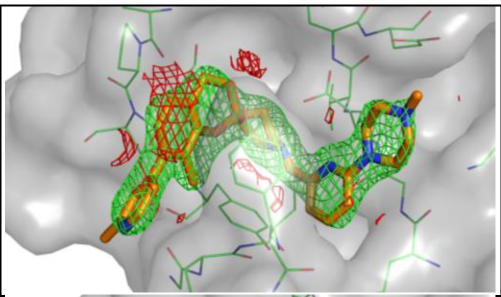	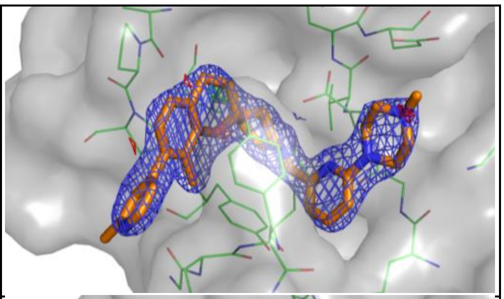
Compound 47<br>9PVI	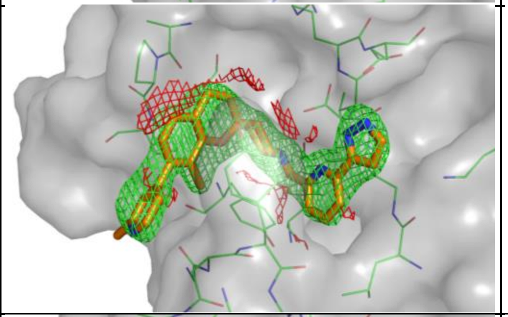	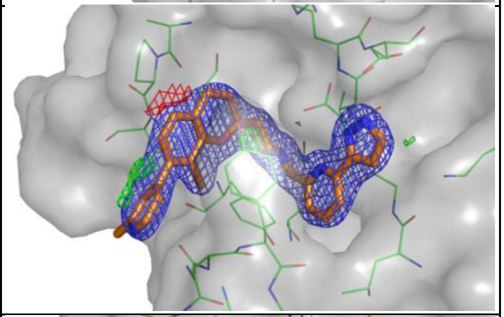
Compound 53<br>9PVK	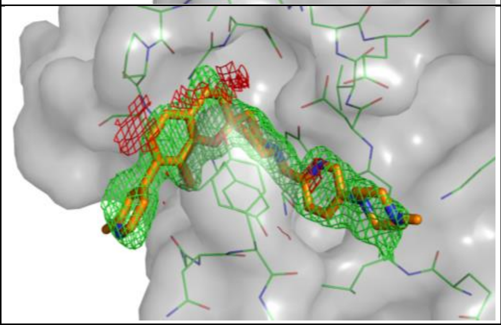	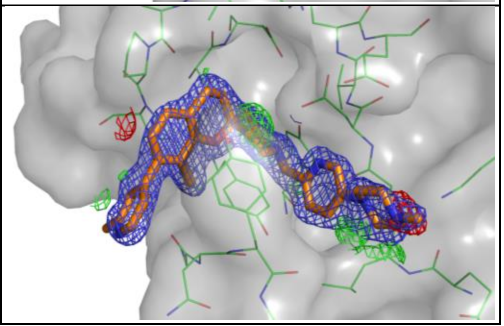

Cellular activity of compounds 44-55

Table S3. Antiviral activity, cytotoxicity and selectivity index for all compounds tested in A549 cellular antiviral assay

Compound[a]	RLKGG IC_{50} (μM)[c]	A549 EC_{50} (μM)	A549 CC_{50} (μM)	A549 selectivity index (SI)[d]
**44**	3.4 ± 1.0	> 10	> 20	NC
**45**	1.9 ± 0.2	7.1	> 20	> 2.8
**46**[b]	0.4 ± 0.1	0.35	19	54
**47**	1.5 ± 0.2	5.1	> 20	> 3.9
**48**	0.6 ± 0.1	0.5	18	36
**49**	0.6 ± 0.1	0.6	20	33
**50**	1.4 ± 0.2	> 10	> 20	NC
**51**	3.5 ± 0.3	> 10	> 20	NC
**52**	40 ± 1.8	> 10	> 20	NC
**53**	0.5 ± 0.2	0.83	> 20	> 24
**54**	1.6 ± 0.2	6.0	> 20	> 3.3
**55**	1.9 ± 0.1	> 10	> 20	> 1.5

[a]Compounds **44-45**, **47-55** were tested with a maximum concentration of 20 μM. [b]Compound **46** was tested with a maximum concentration of 100 μM. [c]Enzyme Inhibition (RLKGG) biochemical data is included for reference. [d]Not calculated (NC)

Initial profiling of Compound 46

A. Whole blood stability

Table S4. The stability results of Compound 46 and control compound propantheline in mouse whole blood

Compound ID	Species	Test concentration (μM)	$T_{1/2}$ (min)	Remaining Percentages (%) 0 min	15 min	30 min	60 min	120 min
Propantheline	Mouse	5	45.53	100.00	89.78	63.08	44.06	16.56
Compound **46**	Mouse	5	> 511.69	100.00	96.47	107.64	99.88	99.88

Note: If % remaining at 120 minutes was greater than 85%, then $t_{1/2}$ will be reported as "> 511.69"

B. Kinetic solubility

Table S5. the kinetic solubility results of compound 46 in 1X PBS pH=7.4

Compound ID	Sample Concentration (mM)	Amount Submitted (mg or uL)	μM	μg/mL	Comments
Compound **46**	10	15	158.5	78.9	Measured solubility is greater than 75% dose concentration, actual solubility may be higher

C. PAMPA permeability

Table S6. PAMPA permeability data of compound **46**

Sample ID	ACCEPTOR	DONOR	C0	Ceq	Pe	%R	Permeability	logPapp
Compound **46**	23.66	146.38	169.06	110.90	5.33E-06	-0.26	+	-5.27
